# Challenges and solutions in the analysis of micro- and nanoplastics down to 500 nm with automated Raman microspectroscopy: suitable filters, accuracy in the detection, identification, and quantification

**DOI:** 10.1007/s00216-026-06567-2

**Published:** 2026-05-29

**Authors:** Isabel S. Jüngling, Lucas Schmitt, Filippo De Franceschi, Paulo A. Da Costa Filho, Lei Lei, Laureen Coic, Nizar Benismail, Stephane Dubascoux, Mark E. Ambühl, Natalia P. Ivleva

**Affiliations:** 1 TUM School of Natural Sciences (NAT, Department Chemistry), Institute of Water Chemistry (IWC), Chair of Analytical Chemistry and Water Chemistry, Technical University of Munich, Lichtenbergstr. 4, 85748 Garching, Germany; 2 Société Des Produits Nestlé S.A. Nestlé Research, Route du Jorat 57, Lausanne, Switzerland; 3 Nestle Quality Assurance Center Vittel, 1020 Avenue Georges Clemenceau, 88800 Vittel, France

**Keywords:** Filter, Microplastics, Nanoplastics, Automation, Raman microspectroscopy, Water samples

## Abstract

Microplastics (1 µm–5 mm) and nanoplastics (<1 µm) are emerging contaminants with potential health implications, requiring reliable detection for risk assessment. Raman microspectroscopy offers combined morphological and chemical information for identifying micro- and nanoplastics (MNPs). This study assesses the feasibility of automated Raman microspectroscopy for detecting and quantifying MNPs down to 500 nm, focusing on suitable filter materials and device precision. Among six tested filters, silicon filters with 1 µm pores (for particles ≥1 µm) and aluminum-coated polycarbonate filters with 0.4 µm pores (Al-PC, for particles ≥500 nm) performed best. They provided strong particle contrast, low background interference, and high Hit Quality Index (HQI) for submicron particles (polystyrene 500 nm beads, median HQI ≈ 91%), outperforming other filters affected by roughness and spectral interference. Automation using open-source software *TUM-ParticleTyper 2* with Random Window Sampling enabled unbiased detection and quantification of MNPs down to 500 nm. Several limitations were noted: illumination settings influenced detected particle number and size recognition, with particles oversized by 0.5 µm ± 0.26 µm; stage precision (~100 nm) affected spectral quality and particle number (<100 particles per window) was critical for achieving >90% correct material identification. Validation with 500 nm polystyrene beads yielded 67 ± 10% recovery relative to theoretical values. Analysis of potable water samples showed predominantly non-plastic particles, with only 0.36 ± 0.13% in the 0.5–10 µm range identified as plastics, 18% of them <1 µm. Further method development, particularly for sample preparation, will be required for broader application to water and food samples. For interest only in particles ≥1 µm, filters with pore size closer 1 µm (e.g., silicon) are recommended. Overall, automated Raman microspectroscopy can quantitatively analyze MNPs down to 500 nm, supporting improved plastic exposure risk assessment.

**Graphical abstract:**

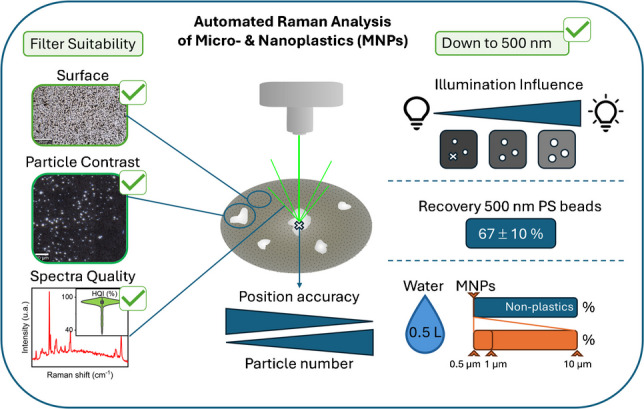

**Supplementary Information:**

The online version contains supplementary material available at 10.1007/s00216-026-06567-2.

## Introduction

Since marine biologist Thompson coined the term microplastics in 2004 [1], public concern about these pollutants and their potential health effects has steadily increased [2–4]. This awareness has driven the need for analytical methods capable of accurately detecting and characterizing microplastics and nanoplastics (MNPs) to assess associated risks. They are assumed to physically affect organisms or act as carriers for harmful chemicals, making reliable data on particle counts, size distributions, and chemical composition essential for risk evaluation [5–7].

Microplastics are solid, water-insoluble plastic particles between 1 µm and 1 mm (or up to 5 mm, depending on definition), while nanoplastics are smaller than 1 µm [8–11]. They are further classified as primary (intentionally produced) or secondary (derived from fragmentation in the environment) [10, 11]. Most MNPs belong to the latter and have been detected in various environmental [1, 12, 13] and food matrices (water [14–22], beer [19, 23–25], milk [25–28], tea [19, 29], sugar [30, 31], table salt [23, 31, 32], honey [25]) and even human tissues (placenta [33–35], lung [36, 37], brain [38, 39]).

While smaller particles (< 1.5 µm) are of particular toxicological concern [40–44], current evidence remains limited and inconsistent [12, 44–46]. This is due to the challenge that MNPs can come from vast sources of various materials and have broad size ranges, shapes, and surface properties. Given that smaller particles are predominant in MNP size distributions [17, 18, 20, 21, 47, 48], the development of sensitive, reliable, harmonized, and standardized analytical methods for their detection and quantification is of great importance.

Conventional methods for microplastic analysis such as Fourier transform infrared (FTIR) spectroscopy and pyrolysis-gas chromatography-mass spectrometry (Py-GC-MS) are widely used for polymer identification but have inherent limitations. FTIR microscopy typically lacks spatial resolution below 10 µm, while Py-GC-MS destroys the particle, preventing morphological analysis [49, 50]. Nanoplastics can be analyzed, for example, by field flow fractionation followed by either offline GC-MS or online Raman analysis [51–54]. However, above-discussed approaches for nanoplastic detection require relatively high plastic particle concentrations in samples and do not enable particle-resolved characterization integrating morphological and chemical information [52]. Raman imaging based on stimulated Raman scattering (SRS) has been used for the detection of MNPs, but at the expense of spectral information [55]. In addition, surface-enhanced Raman spectroscopy (SERS) has been explored to increase Raman sensitivity for micro- and nanoplastic detection by utilizing plasmonic active substrates or nanoparticles to enhance signal intensity. While SERS can significantly improve detection limits, it typically requires specialized enhancing substrates and additional sample preparation steps, which may complicate quantitative particle-by-particle analysis and morphological characterization [56, 57]. In contrast, spontaneous Raman microspectroscopy can theoretically achieve spatial resolution of approximately 300 nm when using a 532-nm excitation laser, depending on the laser wavelength and the numerical aperture (NA) of the objective [58]. In certain studies, resolutions as high as 100 nm have been reported, due to improved signal on the developed filter [59]. Moreover, this technique provides comprehensive spectral information and, when combined with image processing, enables the extraction of morphological characteristics for each detected particle. It has been used in microplastic studies to detect (plastic) particles down to 1 µm on a filter substrate [17, 18, 20]. Nevertheless, particle-by-particle Raman studies in the submicron range (< 1 µm) are lacking. This gap is particularly critical given that small-sized particles are considered the most relevant from a toxicological perspective.

For analysis with Raman microspectroscopy, depending on the sample, a removal of inorganic and organic matrices is required. Various sample preparation strategies, including enzymatic or chemical digestion protocols, density separation, and solvent-based phase systems, have been developed to reduce matrix interferences and minimize false-positive particle identification in complex environmental samples [20, 60, 61]. After the sample preparation, a liquid sample is commonly filtered on a filter substrate to isolate the particles. The filter choice is crucial for Raman-based analysis, as the substrate must provide both high optical contrast for automatic particle recognition and low background signals in the polymer band region(s) [62, 63]. Previous studies have used a range of filters, including aluminum-coated polycarbonate [20], gold-coated carbonate [14], polytetrafluoroethylene (PTFE) [21], anodisc (aluminum oxide) [17], and silicon filters [18]. Ossman et al. (2017) demonstrated that aluminum-coated polycarbonate filters offer preferable properties, such as good optical contrast, low background spectra, and signal enhancement, enabling shorter analysis times and better quality of spectra [62]. Building on these findings, the filters selected in this study were chosen to represent a systematic cross-section of commonly used polymeric and inorganic supports in microplastic studies, including coated and uncoated variants, in order to assess the transferability of favorable substrate properties reported in the literature to nanoplastic detection. Recent developments aimed to apply reflective coatings, such as aluminum, to alternative membrane materials, such as nucleopore PET membranes or aluminum oxide filters, further motivated the inclusion of these filter types. Assessing their performance in terms of particle detectability, spectral interference, and compatibility with automated workflows is essential for advancing method harmonization and standardization.

Manual Raman measurements of small-sized particles are inherently time-consuming and statistically limited due to the low number of particles that can be measured in a reasonable time, especially in the dense particle matrices typical of environmental or food samples [58, 64]. Automated approaches for Raman microspectroscopic analysis (commercial and open-source software) have been developed to increase throughput and reproducibility by integrating image analysis, automated focusing, and spectral identification [17, 18, 65–67]. In addition, due to the large number of non-plastic particles typically present in real samples (environmental, water, food, etc.) in small size range, a subsampling on the filter surface is often required [49, 64, 68]. In this context, the open-source software *TUM-ParticleTyper 2* implemented Random Window Sampling to ensure representative analysis down to 1 µm and allows to detect, quantify, morphologically characterize, and chemically identify particles and fibers directly on filters [67–69]. Optimizing filter compatibility with automated particle recognition and minimizing spectral background are thus key prerequisites for extending the detection range into the submicron range.

The aim of this study was to explore the feasibility of automated Raman-based particle-by-particle approach for the reliable and representative analysis of MNPs down to 500 nm. We focused on suitable filters and on accuracy in the detection and quantification of (plastic) particles. Six different filter types were tested, and filter suitability was evaluated with respect to particle detectability, spectral background, and compatibility with automated analysis workflows. Some challenges faced when applying a fully automated routine are discussed. While *TUM-ParticleTyper 2* was primarily used for the automated approach, the limitations discussed could also be applied to other similar automated procedures. Finally, the applicability of this approach was demonstrated on a water sample, providing a foundation/assessment for reliable detection and characterization of plastic particles down to 500 nm, a size range considered particularly relevant for human health risk assessment.

By systematically assessing filter materials and automated Raman parameters, this study aims to advance towards a harmonized and in the future standardized, high-resolution, and nondestructive analytical workflow for the reliable detection, identification, and quantification of MNPs down to the submicron range. This methodological contribution is expected to increase awareness regarding the challenges when using this technique on submicron particle analysis and improve data comparability, enhance sensitivity for toxicologically relevant particle sizes, and support future risk assessment and regulatory efforts.

## Material and methods

### Filters

Gold-coated polycarbonate (Au-PC) filters (0.4 µm pore size, Ø 25 mm) were purchased from APC GmbH (Germany). Au-PC (0.4 µm, Ø 25 mm), aluminum-coated polycarbonate (Al-PC, 0.4 µm, Ø 25 mm), and aluminum-coated polyethylene terephthalate (Al-PET, 0.8 µm, 25 mm) were obtained from i3 GmbH (Germany). Aluminum-coated aluminum oxide (Al-Al_2_O_3_, 0.4 µm, 10 mm × 10 mm) and silicon (Si, 1 µm, 10 mm × 10 mm and 0.45 µm, Ø 13 mm) were purchased from SmartMembranes GmbH (Germany). Anodisc™ (Al_2_O_3_, 0.2 µm, Ø 13 mm) were obtained from Whatman®. All available pore sizes and corresponding manufacturers are listed in SI, Table S1. The filtration rate for Al-PC (0.4 µm) and Si (1 µm) was measured three times for each filter (100 mL Milli-Q water previously filtered through 0.2 µm PVDF membrane).

### Particles and chemicals

Cryomilled polypropylene (PP) particles sized 0.5–5 µm were obtained from Kiast, Korea (KRM-KBF-PP0.5). A 0.16 mg/mL suspension was prepared and 2 mL filtered on Al-PC filter (25 mm, i3 Membrane GmbH, Germany). Spherical polystyrene (PS)-particles with 500 nm diameter (5 wt%) were purchased from Applied Microspheres GmbH (Germany). A suspension with a theoretical concentration of 1.09 × 10^7^ particles per mL was prepared by dilution. Polyethylene particles sized 0.5–5 µm were obtained from Lab261, Palo Alto, Ca, USA. Micro- and nanoplastic suspensions of polystyrene (PS), polyethylene terephthalate (PET), polylactic acid (PLA), and polyvinyl chloride (PVC) were generated with ultrasonication [70]. PMMA 500 nm was obtained from microParticles GmbH, Germany. Ethanol (1.11727.2500, Supelco, LiChrosolv® Ethanol, ≥ 99.9% (GC), gradient grade for HPLC, CAS: 64-17-5) was purchased from Merck. Milli-Q water was prepared with Milli-Q Integral 5, Merck Millipore (Germany) resistance 18.2 M Ωcm with at least 0.22 μm filter. All chemicals were additionally filtered through a 0.2-μm polyvinylidene fluoride (PVDF) filter (25 mm, Durapore®, hydrophilic, nonsterile, Merck, Germany).

### Sample preparation

For preliminary experiments regarding particle visibility and spectra quality, drops of microplastic suspensions were pipetted on the filter surfaces. Glassware was cleaned with soap, filtered Milli-Q water, and ethanol before use. To remove any potentially remaining plastic particles, the glassware was wrapped in aluminum foil and placed in an oven (L40/11 Nabertherm, Germany) at 500 °C for 6 h. All filtration was carried out in a flow box (ENVAIR eco air V, CARLO ERBA Reagents GmbH, Germany) by vacuum filtration. To reduce the filtration area and allow filtration on the square and hard filters, soft-silicone gaskets were used with an inner diameter of 8 mm (put on top of the filter) and 6 mm (put at bottom of the filter). This was then placed between the vacuum filtration flask and filtration funnel. For concentration determination, 10 mL of filtered MQ-water was added after starting the vacuum filtration, followed by 3 µL of the diluted PS-suspension in triplicate on Al-PC filters. Three 500 mL potable water samples of the same origin were filtered on Al-PC filters. All filtrations were washed with 30 mL of MQ-water and 30 mL of ethanol. Blanks were prepared with the same procedure using MQ-water and ethanol.

### Raman measurements

Two different µ-Raman microscope models from *WITec* (Oxford Instruments, UK) were employed for measurements: the *alpha300 R* and the *alpha300 apyron*, referred to as “alpha” and “apyron,” respectively. Both models utilize confocal Raman imaging and are equipped with a movable stage for automated sample detection and analysis. They are equipped with a 100× (EC Epiplan-Neofluar, NA = 0.9) darkfield capable objective from Carl Zeiss Microscopy GmbH (Germany). All spectra acquisitions were performed with the 532 nm DPPS laser, a grating with 300 lines/mm^2^, and a CCD camera (DU970N-BVF, Andor Technology Ltd., Northern Ireland) as detector. Spectra were recorded in the range of 100–3785 cm^−1^. The “alpha” instrument is equipped with a DFK_X236 camera, and the apyron with a OCV8DFK-33GX camera. For image acquisition, top illumination was set to 100%, gain to 0, and exposure, depending on the microscope, filter, and sample, between 0.009 and 0.002 s. Instrument control, automated image and spectral acquisition, and spectral assignment were carried out using WITecControl SIX 6.2 and WITec TrueMatch/ParticleScout in combination with *TUM-ParticleTyper 2*. Spectral assignment was performed with an in-house built library of bulk materials. Spectral autofocus (s. af.; commonly: *z* = −10 to 15 µm with 0.1 s integration time and a step-size multiplier of 1, exception is the Anodisc filter with instead *z* = −25 to 25 µm and Au-PC with 0.5 s integration time) and the option “Optimize fast” with a SNR-limit of 25 and low signal limit of 50 were applied within the WITec TrueMatch/ParticleScout environment to improve spectral quality and to limit the number of accumulations for automated analysis between 3 and 20. Used integration time, laser powers, and material assignment masks were determined individually for each filter surface and can be found in addition to the spectral autofocus parameters in the results and discussion section in Fig. 2.

For automated measurement, Random Window Sampling implemented in *TUM-ParticleTyper 2* was used to randomly place measurement windows on the filter surface for particle detection and quantification (SI, Fig. S1). The following settings were applied on the “alpha”: For the concentration measurement of the diluted PS-suspension on Al-PC filter, a filter radius of 3750 µm was set, with a window size of 55 µm × 55 µm of which a 50 µm × 50 µm measurement window was set to include all particles between a maximum diameter of 0.5 to 5 µm. The exposure rate was set to 0.0055 s. One thousand two hundred windows were measured and the results extrapolated to the filter area. From that, the concentration per milliliter was calculated. For the water samples, a radius of 3500 µm was set, with a window size of 180 µm × 180 µm (measurement field, 170 µm × 170 µm) with 214 windows measured and another set of 55 µm × 55 µm (measurement field, 45 µm × 45 µm) detecting particles with a maximum diameter between 0.5 and 10 µm with at least 600 windows was measured. The exposure rate was set to 0.007 s.

### Particle size verification

For size verification of the polydisperse PP sample, an image was taken on WITec apyron, objective 100×, NA = 0.9 close to a recognizable spot, with an exposure of 0.004 s, illumination 100% and gain 0, image size 200 µm × 200 µm and analyzed with our in-house Software *TUM-ParticleTyper 2* and the Microscopes Software WITec ParticleScout (setting: auto). The same spot was also analyzed with a Sigma 300 VP field emission SEM (FE-SEM, Carl Zeiss AG, Germany). An acceleration voltage of 10 kV, a working distance of 8.8 mm, and a magnification of 1.19 K× were employed for image acquisition with an SE detector. Particles were colored in by hand and analyzed with *TPT-2* and ImageJ. The size for the PS beads was obtained from the results from the concentration measurements on the Al-PC filter measured with the WITec alpha system. The results were compared to PTA results and the particle size also checked with SEM.

### Particle measurability/stage precision

In total, six measurements were performed for each Raman microscope (three with positioning determined with *TUM-ParticleTyper 2* and three with WITec ParticleScout). For each measurement, a 200 μm × 200 μm window containing approximately 200 particles was analyzed. The particles used were cryomilled polydisperse PP particles with a size range between 0.5 and 5 μm. For each Raman spectrum, the same settings for spectral data acquisition were used: ten accumulations with an integration time of 0.5 s, utilizing a 532 nm laser set to a power of 3.5 mW. A 100× magnification objective (NA = 0.9, WD = 1 mm) was employed. No spectral autofocusing was applied, with a fixed *z*-axis position at 0. After the measurements were completed, the recorded spectra were compared with in-house databases to assign a fitting material to each particle’s spectrum. The HQI value was set to 0, and each spectrum was evaluated and confirmed by an expert. Particles that were not recognized as PP were remeasured individually by hand to confirm or correct the initially determined material. If a different spectrum or material was identified upon remeasurement, the particle was marked as false. After 50, 100, 150, and 200 measured particles, the percentage of correctly automatically assigned particles has been determined.

### Particle tracking analysis (PTA)

The ZetaView 230 from ParticleMetrix GmbH (Germany) was used to determine the hydrodynamic diameter and concentration of the PS-suspension. The suspension was injected into the system in light scattering mode. A triplicate was measured. Before measurement, a system validation was performed with 100 nm polystyrene size standard.

### Flow cytometry (FCM)

The flow cytometry analyses were carried out with a Cytek Aurora (Cytek Biosciences Inc., USA) equipped with five solid-state lasers (355 nm, 405 nm, 488 nm, 561 nm, 640 nm) and with an enhanced small particle (ESP) detection option that enables a higher resolution for nanoparticles down to 70 nm. The raw unmixed FCS files produced by the instrument were analyzed offline with the software FCSExpress version 7.26.0020 (DeNovo Software inc., USA). Five hundred microliters of each sample has been acquired at a high flow rate (100 µL/min) and with an arbitrary electronic threshold on SSC = 500. Side scatter height on the 405 nm laser (SSC-H) and forward scatter height (FSC-H) were used to enhance the displayed resolution of the beads on the plots. The first selection of the PS beads was done (SI, Fig. S2, plot A) to eliminate the debris present in the preparation. Additional assessments were made to enumerate the PS beads that aggregate together forming doublets, triplets, quadruplets, etc. (SI, Fig. S2, plot B), and the total number of beads in suspension was corrected considering these aggregates.

## Results and discussion

### Filter selection

Filters from four different manufacturers were procured, and in total six different filter types were investigated: Au-PC, Al-PC, Al-PET, Anodisc™, Al-Al_2_O_3_ and Si filter. All of them can be procured down to at least a pore size of 0.45 µm or smaller (SI, Table S1).

One of the aims of this work was to investigate suitable filter substrates for the analysis of microplastics down to 1 µm and suitable filter substrates for the analysis of particles down to 500 nm. All evaluations are performed with the particle recognition provided by *TUM-ParticleTyper 2* and image acquisition, Raman measurement, and material assignment with the WITec software.

The following four aspects must be considered when choosing a filter substrate for automated analysis:

1. Pore size: The pore size needs to be small enough to retain all particles of the size range of interest. In the case of analysis of particles down to 500 nm the filter pores should be smaller than this.
2. Particle visibility: To allow good particle detection (automatically or manually) particles must be visible on the filter surface. For microplastic particles, images are commonly obtained in darkfield, as the plastic particles usually appear as white spots on a dark surface. The larger the contrast of particles towards the filter surface, the better the detection by an automated program. In this regard, it is important that the substrate surface is unstructured and smooth. If this is not the case, the filter structure may be recognized as particles itself and potentially obstruct the visibility of smaller particles. To a certain degree, *TUM-Particle Typer 2* can reduce the background structure, especially for uniformly distributed, small-sized features, such as filter pores or residual reflections, resulting in a dark background, enhancing the particle contrast further [67].
3. Raman signal and background: For the analysis of tiny particles, it is crucial that the filter substrate does not exhibit interference Raman bands from the filter and/or (strong) fluorescence background within the spectral ranges relevant for identification of plastic types (590–1770 cm^−1^; 2690–3200 cm^−1^). Especially filters with a high number of Raman bands in the region of interest could potentially compromise the quality of the measurements.
4. Automated material recognition/Spectra quality: Automated material assignment allows a great reduction in workload and faster results. For the program, to confidently assign the correct material, a high-quality spectrum is needed. Suitable laser power, accumulation number, integration time, and spectral mask must be chosen to obtain a high Hit Quality Index (HQI), if a global HQI value is used. The HQI should reach for the settings described in methods for polymer mixtures at least a value of 45 to ensure a correct automatic material assignment of over 70% (see SI, Fig. S3). With the use of the ‘Optimize fast’ feature of the WITec systems, the number of accumulations necessary can be dynamically adjusted to lower numbers (minimum 3 accumulations) if the spectrum quality reaches a set value (e.g., SNR (signal-to-noise-ratio)–Limit of 25) reducing the overall measurement time.

As the Raman signals from the filters play an important role, filters such as nitrocellulose, polyvinylidene fluoride (PVDF) or PTFE without coating were excluded due to their known strong Raman bands in the region of interest.

#### Pores

**Fig. 1:**
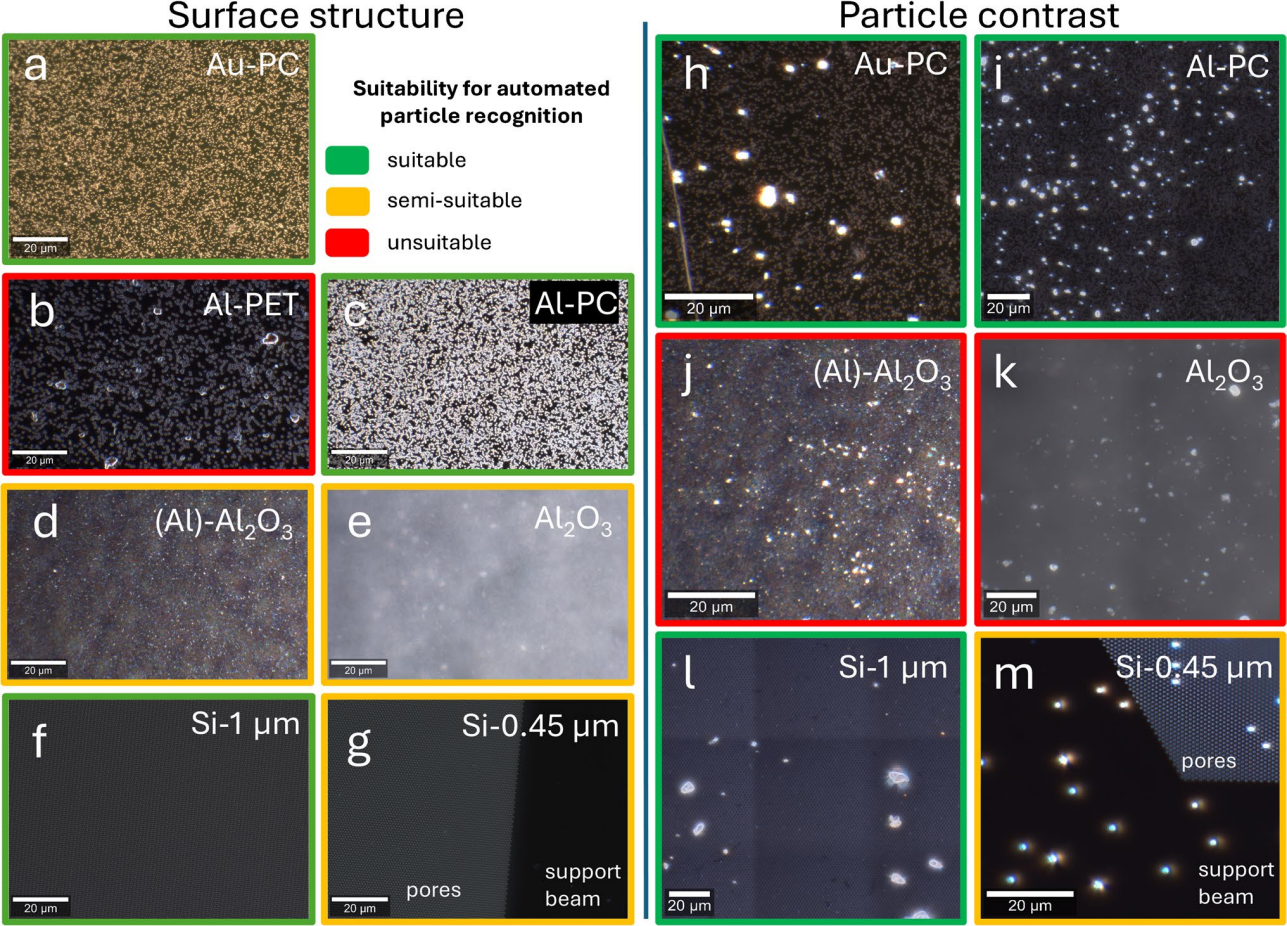
Optical microscope images acquired with a 100× magnification objective with darkfield illumination of various filter surfaces without (**a**–**g**) and with microplastic particles (**h**–**m**). Depicted are gold-coated PC membrane (**a**+**h**), aluminum-coated PET membrane (**b**), aluminum-coated PC membrane (**c**+**i**), aluminum-coated Al_2_O_3_ (**d**+**j**), Anodisc Al_2_O_3_ (**e**+**k**), Si-1 µm pore size (**f**+**l**), and Si-0.45 µm pore size (**g**+**m**). Latter images (**g**+**m**) depict the pores (gray area) and the support beams (black area) of the Si-0.45 µm filter. The surfaces of **a**, **c**, and **f** were deemed suitable (green border), **d**, **e**, and **g** semi-suitable (yellow border), and **b** unsuitable (red border) for automatic particle detection. Good particle contrast suitable for automated analysis is obtained with **h**, **i**, and **l**, while **m** is semi-suitable due to necessary code adjustments and **j** and **k** are unsuitable for polydisperse samples. All scale bars show 20 µm

The flexible track-etched polycarbonate membrane filters show irregularly distributed pores (Fig. 1a–c), with occasionally larger pores due to the accumulation of nearby pores, risking possible loss of small-sized particles. The Al_2_O_3_ and Si membranes on the other hand are more rigid and more brittle with the Si filter being much more stable than the Al_2_O_3_ filters. The pores of Al-Al_2_O_3_ and Si filters are distributed in a grid-like structure. For the Si-0.45 µm filter, the areas with pores are distributed in a honeycomb structure to support the fragile pore grid with the support beams being approximately (100 µm) wide (Fig. 1g, m). Although Si-1 µm filter got a pore size too large for measuring particles down 0.5 µm, it has been included in the study as it has not been previously described in its properties regarding MNP analysis down 1 µm to our knowledge, though they have been used for MP investigation [18].

#### Au-PC

Gold-coated polycarbonate membranes of two different manufacturers have been investigated, and they can be procured in various pore sizes down to 0.1 µm in various diameters (25 mm, 47 mm). The commercially available membranes are usually coated with a 40 nm gold layer. As they gave similar results, they are not presented separately.

**Fig. 2:**
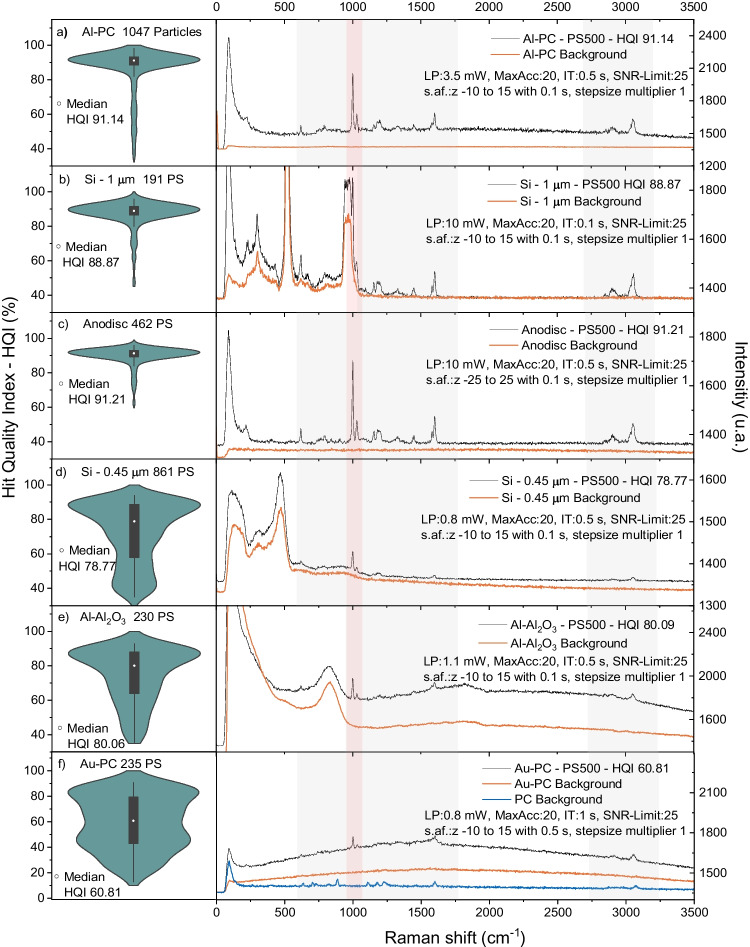
Left: Violin plots of HQI distributions for automatically measured 500 nm PS particles on different filter surfaces. Plot width indicates kernel density; box plots show interquartile range (25–75%), whiskers extend to 1.5× IQR (interquartile range), and the dot marks the median. Measured particles number listed on top. Right: Corresponding Raman spectra (black) represent PS spectra at the median HQI, with background filter spectra (orange) shown for comparison. Gray underlay marks the spectral mask used for material identification. Red underlay emphasizes characteristic PS-double band. All spectra were obtained on WITec alpha 300R with a 100× magnification objective. The individual settings for “Optimize fast” option are given in the legends with laser power (LP), max. accumulations (MaxAcc), integration time (IT), and spectral autofocus (s.af.)

The surface, when observing under the light microscope, is smooth and unstructured (Fig. 1a). As already described by Schymanski et al. and Oßmann et al*.* [14, 62], darkfield illumination produced well-contrasted white particles for the filtered plastic particles (Fig. 1h), well detectable with the automatic program. Similar to the observation reported by these authors, a rather large fluorescence background could be observed in the Raman spectra. Occasionally even spectra of the PC membrane were obtained. Due to warming of the gold surface, particles burn when measuring at high laser power with a 532 nm laser. It was possible to obtain spectra of the 500 nm PS beads only at a laser power below 1 mW (0.8 mW, max accumulation 20, integration time 1 s) with a broad HQI value distribution also in the region below 45%. They were often mixed with soot bands, which makes it not feasible for automatic spectra assignment. Additionally, the spectral autofocus integration time had to be raised from 0.1 to 0.5 s to find the best measurement z-position, which further increases the overall measurement time (Fig. 2f). To obtain high-quality spectra of all beads visually detected, this would likely have to be raised even further.

As such, while Au-PC might be suitable for the measurement of larger-sized particles (as shown by Schymanski et al. [14] and illustrated in Fig. S4) or simple particle size determination, it is not suitable for the MNP automated analysis down to 0.5 µm. 

#### Al-PET

Aluminum-coated PET polycarbonate filters were excluded after investigation under the microscope due to their pronounced surface structure. The surface is neither smooth nor unstructured, leading to extensive false-positive particle recognition and severe difficulty in differentiating between particles and the filter surface. Performing particle recognition on a non-treated blank Al-PET filter (given filter radius 9750 μm) resulted in over 6000 recognized particles, most of which can be identified as filter structures. These large structure-induced features are likely to obscure or cover smaller particles and, therefore, critically impair the reliable detection of sub-micrometer particles. Closer investigation using SEM resulted in the observation of “craters” in the filter membrane, which look like particles under the light microscope (Fig. 1b, Fig. S5). While other filters, such as Anodisc or aluminum-coated aluminum oxide, also exhibited particle-like background features, these predominantly occurred at much smaller sizes, closer to the sub-micrometer range, and in fewer numbers, and were therefore less obstructive for particle detection. Furthermore, PS beads could still be recognized on Anodisc or aluminum-coated aluminum oxide filters by appropriate adjustment of focus position and illumination settings, whereas the crater-like structures on Al-PET remained detectable and indistinguishable from particles under all tested conditions. As the observed surface characteristics directly interfere with automated particle recognition, the Al-PET filter was deemed unsuitable for further investigation and was excluded from subsequent analyses. In addition, PET is one of the most used plastics for packaging and would need to be excluded during analysis due to potential contamination through the membrane.

#### Al-PC

Aluminum-coated polycarbonate membranes are from i3 company. They can be requested in various pore sizes down to 0.1 µm. In total, five different types of aluminum coating were tested. Their optical and spectral properties differ not much, and only the latest developed filter is presented here. They are flexible, coated with 100 nm of aluminum, showing a very smooth and unstructured surface and a good contrast between particles and surface background (Fig. 1c, i). No polycarbonate background spectrum was detected. The background signal in the Raman spectrum is low in the region of interest (low noise and no fluorescence), and no interference bands are visible. The characteristic bands of plastic material are well visible when measuring plastic particles. Spectra of the PS 500 nm beads were acquired at a fixed laser power (3.5 mW, determined in preliminary laser power tests) to avoid particle burning, with the majority of spectra yielding HQI values above 80% at 0.5 s integration time and 20 accumulations (median HQI 91.14%, Fig. 2a). Spectral autofocus was found to be necessary as it is not possible to fully flatten the filter membrane lower than ± 5 µm with the filter holders described by von der Esch [65].

#### Al-Al_2_O_3_

The Al-Al_2_O_3_ filters from *SmartMembranes* company are rigid, cut as a square and mounted on a different filter holder. They have a side length of 10 mm and a pore size of 0.4 µm. They are of gray-metallic color and slightly reflective. The pores are not randomly distributed as for the flexible membrane filters but form a grid. Additionally, the Al-Al_2_O_3_ filter is very brittle and prone to breaking. Although the Al-Al_2_O_3_ filter shows some particle fragments, only slightly over 100 particles in the 10–40 µm size class were detected. In the smaller size range more particles are found, which can hinder automated analysis (Fig. 1d). A SEM image showed them to be accumulations of the Al-coating (SI, Fig. S5). They were deemed semi-suitable for the automated particle detection, as the accumulations could be mistaken as particles, but were low in number. While PS spheres were identifiable and detectable with automated particle recognition, polydisperse PP particles were difficult to differentiate (Fig. 1j) from the filter surface, since they did not show a good contrast. With this the Al-Al_2_O_3_ was deemed unsuitable for automated particle recognition. The spectral background of the filter shows a broad band between 700–900 cm^−1^, which has to be excluded for material recognition. It was possible to perform automatic measurements on the PS beads, with a laser power of 1.1 mW, obtaining a median HQI value of 80.06% for an integration time of 0.5 s and maximum accumulations of 20. The HQI distribution is spread to lower HQI values (Fig. 2e). Due to the above reasons, primarily related to particle recognition, we excluded the filter for further investigation.

#### Anodisc (Al_2_O_3_)

The Al_2_O_3_ filter‚ Anodisc filter, is round with a white color and very brittle with a pore size of 0.2 µm. A filter support similar to the polycarbonate filters was created for them. In areas with a high concentration of PS 500 nm beads, they were possible to be clearly differentiated from the filter surface and detected by automated particle recognition. But in areas with no beads or irregularly shaped PP particles, it was difficult to differentiate between filter surface imperfections and particles (Fig. 1e, k; SI, Fig. S6). The automatic video focus was often not able to focus on the filter surface, and we observed a *z*-axis difference of up to ± 25 µm, which increased due to thermal expansion when measuring with the laser. As such, it is necessary to increase the focus stacking of the video image and spectral autofocus range, which significantly increases measurement time. The signal in background spectra was low with no significant bands in the area of interest, providing good Raman spectra of reference plastic particles. It was possible to measure the PS beads with a laser power up to 10 mW obtaining in majority high HQI values at a median value of 91.21% with an integration time of 0.5 s at 20 accumulations (Fig. 2c). While their spectral properties are very good, it has been decided to exclude them for further investigation due to their limitations regarding the focusing issue, issues in automatic particle detection, and thermal expansion. If the windows were not randomly placed, but a large-sized window, with the use of a true surface module, these might be of interest for other users [17]. But in this case, other limitations discussed in (2) Automated analysis—challenges will have to be considered.

#### Si-1 μm

The Si-1 µm filters procured from *SmartMembranes* company are rigid and cut into 10 mm × 10 mm squares. The advantage of the Si filter is that, unlike PC membrane–based filters, it is possible to clean and even reuse the filters by burning plastic particles in the oven, ensuring a fully plastic-free filter for analysis. It is, in general, very stable, also regarding extensive chemical sample preparations. The surface is unstructured and very flat and can be placed evenly below the microscope (Fig. 1f). Depending on the sample, it is possible not to use the spectral autofocus to save time, though in general it is recommended to use it to gain the best spectral quality. The particle contrast is good and suitable for automated particle detection (Fig. 1l). While some very bright particles show a surrounding “halo,” this effect can be reduced to a minimum when choosing appropriate illumination settings. The only downside is the prominent Si band at around 520 cm^−1^ and broad band around 920 cm^−1^, also visible when measuring large (> 5 µm) plastic particles (Fig. 2b, SI, Fig. S6). There are two options to circumvent that for the material assignment. The WITec Software offers the option of the subtraction of a Si background spectrum and/or only bands, excluding any in the region of the bands, are included in the spectrum evaluation. Excluding the peaks and subtracting the background spectrum were shown to provide the best HQI values. PS-500 nm were detected on the cross sections of the filter grid, with HQI values in the majority around the median value of 88.87% with an integration time of 0.1 s, 20 maximum accumulations, with a laser power up to 10 mW before burning of particles (Fig. 2b). For analysis down to 1 µm, this filter would be a good and adequate option.

#### Si-0.45 μm

Si-0.45 µm, also procured from *SmartMembranes* company, is a novel filter developed with the pores arranged in “pods” in a honeycomb structure. These resulted in some challenges as it does not meet the requirement of an unstructured surface, as we have some fields of view with pores visible and the support beams visible (Fig. 1g). The particles are not randomly distributed as the majority of particles gather in the pods. The Fourier transform filter, usually used to reduce pore noise [67], resulted in erasing the particles for those fields of view and had to be removed from the code to analyze the images. By adjusting the illumination settings, it was possible to get a good particle contrast to the filter surface (Fig. 1m). Focus stacking and spectral autofocusing are a necessary requirement as the pods show height differences of ± 10, and even in small images (55 µm × 55 µm), the surface was never fully flat. In contrast to the Si-1 µm filter, no disruptive background spectrum was found, except in the region of 0–500 cm^−1^ which is outside the region of interest for polymer bands. A laser power of 0.8 mW already produced spectra with HQI values above 45 with 0.5 s integration time and as low as 3 accumulations for PS-500 nm beads. With automated measurement parameters set at 0.8 mW, integration time 0.5 s, and max accumulations 20, a median HQI value of 78.77% was obtained (Fig. 2d). The values are more spread to lower values in comparison to the Si-1 µm filter. Higher laser powers did not appear to burn the PS- 00 nm particle but appeared to push the particle into the grid. They were still measurable but with a much lower intensity and were barely visible in the video image; as such, a reanalysis of the same filter area is not recommended. If, for the future, it might be possible to generate these filters without the honeycomb structure, they would make a good chemically resistant filter support material for the analysis of particles in the submicron region.

For further investigation and next evaluations in this study, with the focus on particles below 5 µm and down to 500 nm, it has been decided upon the Al-PC with a pore size of 0.4 μm filter as the preferred filter material, due to its optical (flat, unstructured surface and good particle contrast) and spectral properties. Measured PS beads resulted in a high median HQI value (91.14%) with 25–75% of the values lying between 89 and 93%), although the Si-1 µm filters also showed promising results for particles down to 1 µm. While the filtration rate was not the primary selection criterion for determining the automated Raman compatibility, it is important for practical sampling later on. The measured flow rates were 11.1 ± 1.2 mL/min for the Al-PC (pore size 0.4 µm, filtration area 28 mm^2^ (ø 6 mm), 40.5 ± 0.3 mL/min for filtration area 132 mm^2^ (ø 13 mm) and 15.3 ± 5.7 mL/min for the Si-1 µm (filtration area 28 mm^2^ (ø 6 mm)) (see SI, Table S2).

**Fig. 3:**
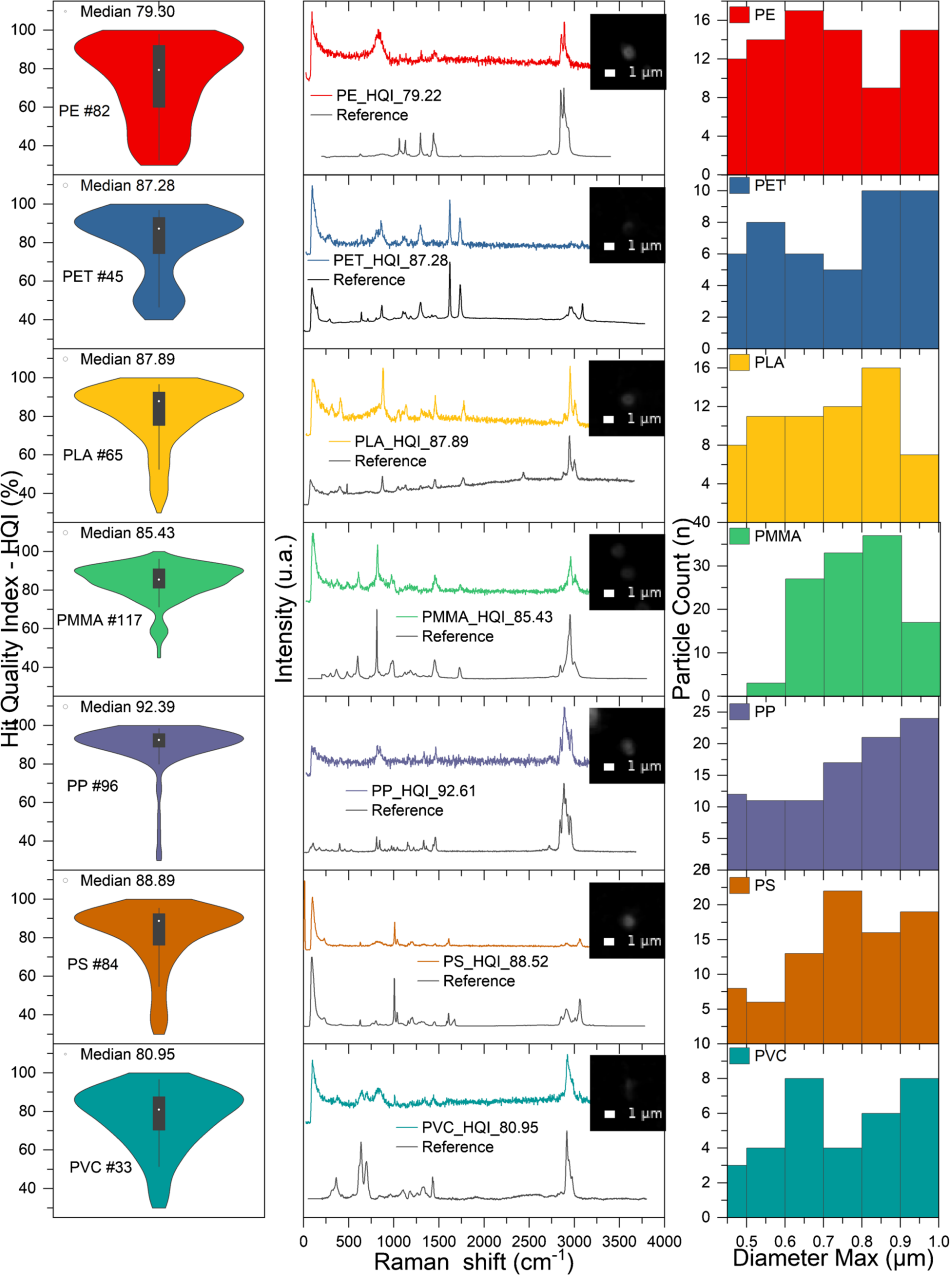
Left: Violin plots of HQI distributions for plastic particles (PE, PET, PLA, PMMA, PP, PS, PVC) automatically measured on Al-PC filter with measurement parameters used in Fig. 2 for PS beads. Plot width indicates kernel density; box plots show interquartile range (25–75%), whiskers extend to 1.5× IQR (interquartile range), and the dot marks the median. Measured particle numbers are listed with a number sign. Middle: Same-colored Raman spectra represent plastic spectra at the median HQI or as close as possible. The particle image is of the corresponding measured particle. The database spectra obtained from bulk measurements (black) are shown for comparison. Left: Histogram of the size distribution of the measured particles

For the reliable analysis, it is important to determine whether other polymer types and particle shapes can be detected similarly to the PS beads. To verify the applicability of the method to different polymer types and non-microspheres (irregular shapes), we used PE, PET, PLA, PMMA, PS, PP, and PVC particles. They were measured on an Al-PC filter with the determined settings from the PS-bead comparison (Fig. 2). The particles were in the size range below 1 µm to the lowest possible size detectable with the *TUM-ParticleTyper 2* of 0.46 µm. As shown in Fig. 3, although the HQI distributions exhibit different shapes depending on the polymer type, all median HQI values exceed 79%. It indicates that nanoplastics can be reliably and automatically assigned using the bulk spectral library.

The spectra show the characteristic bands of the corresponding bulk materials, though their overall quality is lower. This observation is consistent with previous reports showing that nanoplastics exhibit the same characteristic peaks as their bulk counterparts, with only minor modifications in peak position, width, and intensity [71]. When comparing the results of the polydisperse, fragmented PS to the PS beads, a slightly lower median HQI value (88.89% vs 91.14%) and a wider distribution are observed. This indicates that environmentally relevant particles are likely to exhibit a lower HQI than model materials. In real samples, additional factors such as aging may further modify the spectral features.

### Automated analysis—challenges

#### Particle recognition

**Fig. 4:**
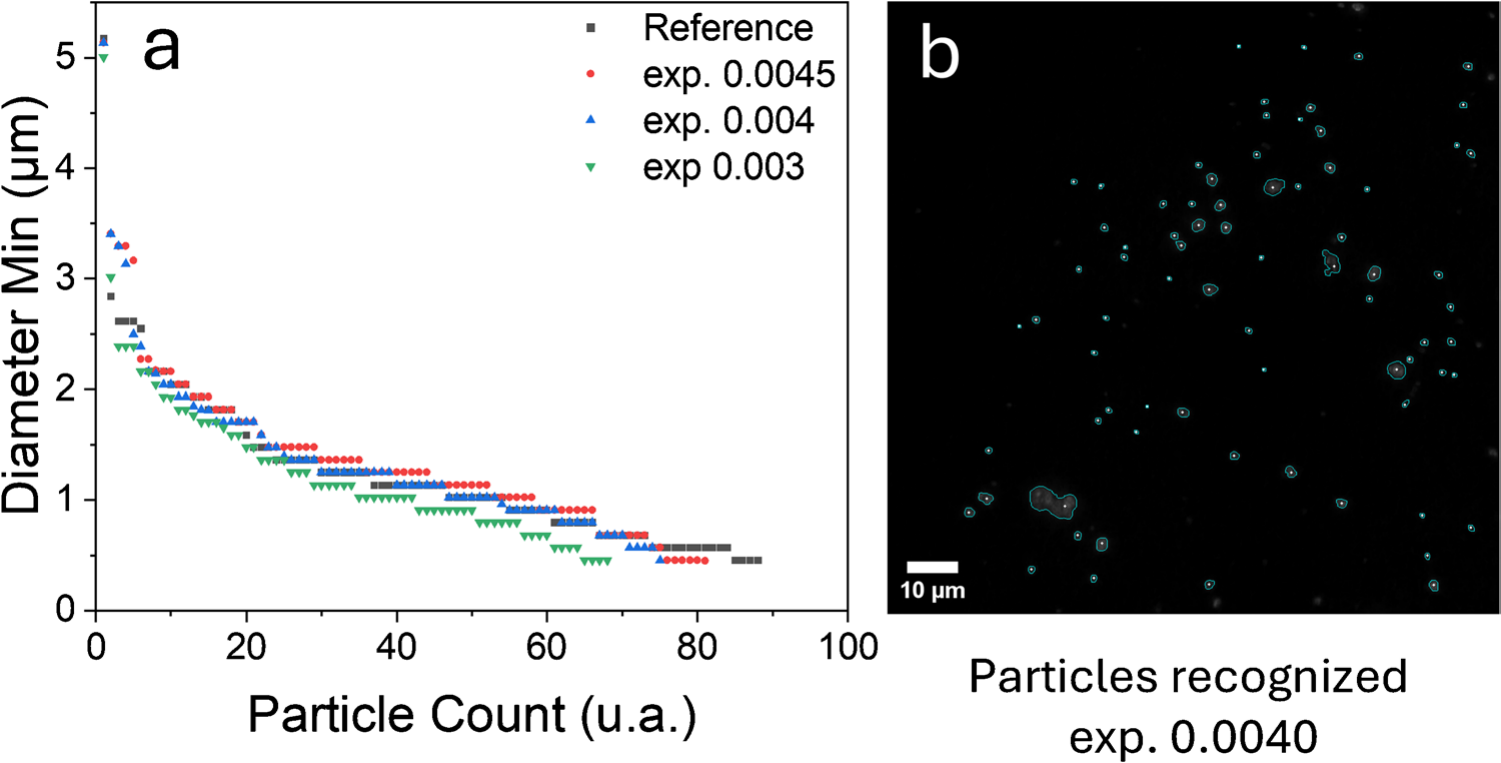
**a** Particle sizes and numbers recognized at various illumination settings (top illumination 100%; gain 0; exposure 0.0045 s, 0.004 s, or 0.003 s). The illumination settings shown here are not intended as a systematic optimization but serve to illustrate the influence of camera exposure on particle recognition and apparent particle size. Black displays the sizes obtained from a colored-red reference image. Particle numbers are more accurately recognized with brighter illumination settings, but particles are more enlarged. **b** Image with exposure 0.0045 s used for particle recognition in **a**, with the best match regarding the reference. Recognized particles are bordered in blue and the measurement points are marked with a white dot

First, regarding particle recognition, it has to be noted that the quality of the acquired image plays an important role in the size recognition of the particles. In the case of the microscope cameras used in the study, with the use of a 100× magnification objective (NA = 0.9, WD = 1 mm), it is limited to a nominal resolution of 0.11 µm/pixel (depending on image size), and the minimum size of the maximum diameter of bright particles we can determine with our software is 0.46 µm. The software itself only differentiates between bright and dark pixels when recognizing the size. This means the illumination settings of the acquired camera image play a major role in the size at which the particles are detected. The settings given here as an example are not intended as a systematic optimization, but to showcase the effect of a parameter on particle recognition and detected particle size. Gain and top illumination were kept constant to isolate the effect of image-based illumination parameters for particle recognition. With brighter settings (e.g., exposure 0.0045), the particles appear larger, but if the settings are too low (e.g., exposure 0.003) and the image is too dark, low contrast particles might not be recognized anymore (Fig. 4a, b). As such a balance needs to be found (e.g., exposure 0.004). The illumination settings required differ greatly between microscope devices (SI, Fig. S7a, b), used objectives (two 100× magnification objectives can require different illumination settings on the same microscope), filter support and sample type. As such, the settings mentioned in this paper are unique to our devices and serve solely as guidelines.

**Fig. 5:**
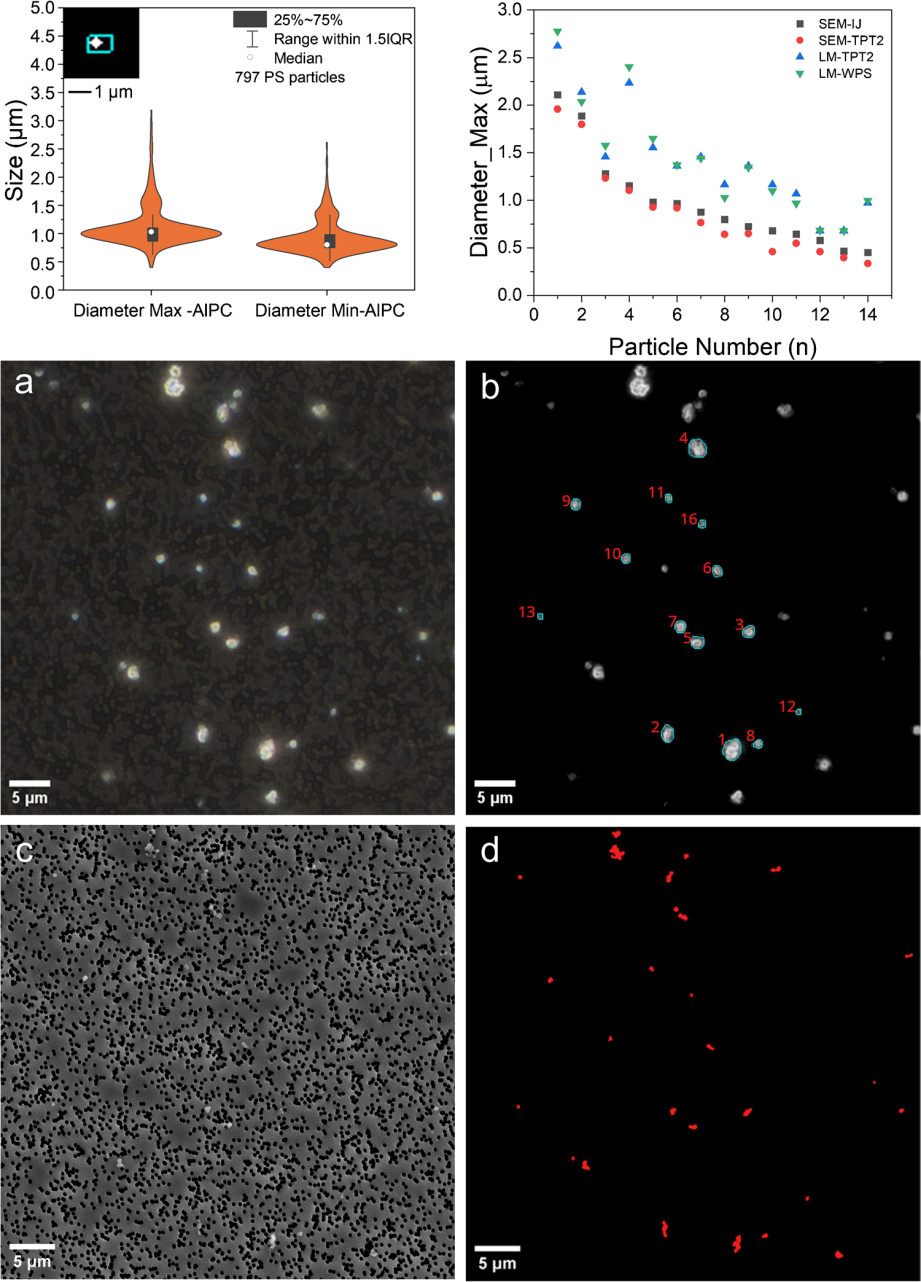
Upper left: Violin plots of particle size distributions of diameter max and diameter min for automatically detected and measured 500 nm PS particles on Al-PC filter with an exposure of 0.0055 s. Plot width indicates kernel density; box plots show interquartile range (25–75%), whiskers extend to 1.5× IQR, and the dot marks the median. Measured particle numbers listed in legend. Image of median size recognized PS-bead in upper left corner of violin plot graphic. Upper right: Detected size of PP particles with light microscope and SEM, particle size calculated with either *TUM-ParticleTyper 2* (TPT2), WITec ParticleScout (WPS), or ImageJ (IJ). **a** PP particles on Al-PC are taken with darkfield light microscope (exp. 0.00045). **b** Assigned particle numbers for particles recognized with light microscope and SEM. **c** SEM image of the same particles. **d** Colored in found particles of SEM image for particle size recognition with either TPT2 or IJ

When measuring PS beads 500 nm on the Al-PC filter, a bimodal distribution was observed. The majority of PS beads 500 nm particles were not detected at their nominal diameter of 500 nm, but their maximum diameter was detected as 1.1 ± 0.4 µm, with the minimum diameters being detected as 0.9 ± 0.3 µm (Fig. 5a). In contrast, PTA measurements (520 ± 13 nm), as well as SEM images (SI, Fig. S8), confirmed the expected radius of approximately 500 nm. Some even larger particles were found, which could be attributed to agglomeration of PS particles that cannot be deagglomerated at this size level due to the spatial resolution (SI, Fig. S8). When taking images with a light microscope, spheres at this resolution level (0.11 px/µm) are no longer detected as circular objects but appear rectangular in the acquired image due to pixel discretization (Fig. 5, left). On average, the PS particles (excluding particles above 1.3 µm, attributed to the second mode of distribution) were detected 0.48 ± 0.13 µm too large. This systematic size overestimation can be quantitatively explained by the combined optical and algorithmic limitations of the imaging system. The spatial resolution relevant for particle size determination is defined by the video imaging path. Using the Rayleigh criterion *d*≈0.61λ/NA, and assuming visible illumination in the range of approximately 450–650 nm with NA = 0.9, the diffraction-limited lateral resolution is approximately 0.30–0.44 µm. Particles near this size are therefore broadened by the point spread function of the optical system. In addition, the camera resolution of 0.11 µm/pixel introduces discretization effects. In darkfield imaging, edge-enhanced scattering produces intensity halos that extend beyond the geometric particle boundary. During adaptive thresholding, these halo regions are classified as part of the particle, leading to an outward displacement of the segmented boundary. Because boundary placement is quantized at the pixel level, this results in diameter increments of approximately 0.22 µm per one-pixel radial shift (0.11 µm/pixel) in case of symmetrical boundary shift, while a two-pixel shift corresponds to approximately 0.44 µm. A symmetrical boundary shift is not always observed, as local intensity gradients and pixel-grid discretization can lead to direction-dependent displacement of the segmented contour (Fig. 5, left). *TUM-ParticleTyper 2* uses prior to adaptive thresholding (used for boundary segmentation) gray-value morphological opening with a 5 × 5 kernel (effective radius 2 pixels) which smooths intensity gradients and can modify object boundaries by up to 0.22 µm in radius [67]. The segmentation algorithm itself is deterministic; repeated analysis of identical images yields identical size values, indicating that the software does not introduce stochastic variability (data not shown). Comparable size overestimations were observed when using WITec ParticleScout for particle evaluation, although the internal segmentation procedures of this third-party tools are not fully accessible. The dominant software-related contribution therefore arises from pixel-based boundary placement and pre-threshold smoothing inherent to threshold-based segmentation procedures rather than from numerical instability. Together, diffraction-induced optical broadening (~ 0.30 to 0.44 µm) and segmentation-related discretization (~ 0.2 to 0.4 µm) quantitatively account for the observed mean size overestimation of 0.48 ± 0.13 µm for PS beads. To evaluate whether this systematic size overestimation also applies to more complex and realistic samples, we next investigated polydisperse and non-spherical PP particles. An experiment with more realistic, polydisperse, and not uniformly shaped PP particles was performed. The sizes detected from the images of the light microscope were compared with the sizes detected from the same particles with scanning electron microscopy (SEM). Not all particles visible with the light microscope of the Raman instrument were visible with the SEM, due to “swallowing” of e-ray through filter pores (Fig. 5a, c). Overall, the light microscope, independent of the software used to detect the particles, estimates these not uniformly shaped particles to be larger by 0.5 µm ± 0.26 µm (Fig. 5, right). Similar to the PS spheres, some particles are detected closer to their actual size (e.g., *n* 12, 13) than others (e.g., *n* 10, 11) (Fig. 5 right, b). This is again dependent on the contrast of the particle towards the filter surface and its “halo” casting, as it affects the smoothing and positioning of the boundary placement. Real samples are expected to contain a multitude of different materials with different optical properties. It must be kept in mind that when giving particle numbers in those size ranges obtained by Raman microspectroscopy, the sizes in these ranges are not clearly defined and it is not possible to make a clear distinction between submicron and micron-sized particles. This might cause overestimation of plastic particle numbers above 1 µm, when using filters with a pore size below 1 µm. Particles detected as below 1 µm can be classified with more confidence as nanoplastic particles.

#### Measurability of particles

**Fig. 6:**
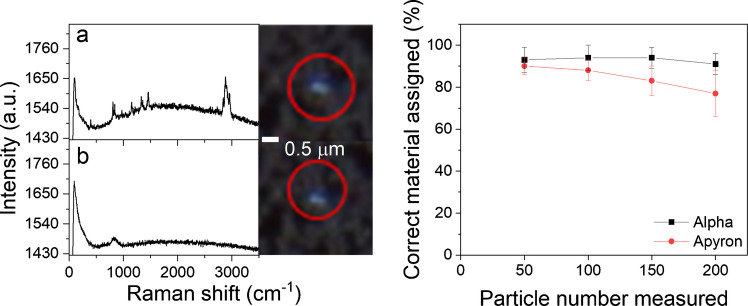
Left: Example of a small-sized PP particle on Al-PC filter, with respective spectra depending on the center of laser focus (red circle). The fully centered (**a**) particle resulted in a recognizable PP spectrum, while off-center (**b**), no assignable spectrum was obtained (spectra acquisition: integration time 0.5 s, accumulations 10, laser power 3.5 mW, 100× objective, NA = 0.9). Right: For different microscopes’ mean values of the number of correctly automatically assigned material spectra in dependence on the number of particles already measured. The rate of decrease in accuracy over time is device dependent. Most particles were detected as being below 2.5 µm in diameter, with approximately 40% below 1 µm

Another critical consideration is stage position precision, particularly for small-sized particles. Although it is in general possible to measure particles as small as 500 nm, they must be precisely centered in the laser focus to obtain high-quality spectra (Fig. 6, left a). The stage movement precision given by the manufacturer is 100 nm. Small deviations already show a large effect in spectra acquisition (Fig. 6, left b). A polydisperse PP sample with the main size range being detected between 0.5 and 2.5 μm, with approximately 40% of the particles being recognized as below 1 μm, was used to determine the stage precision for small-sized particles. Following automatic material assignment, each spectrum was individually reviewed and classified as PP or non-PP. Particles identified as non-PP were subsequently remeasured manually to confirm accurate material assignment. It was found that the ability to acquire correctly assignable material spectra deteriorates with increasing particle number when measuring a large number of particles in one window and is device-dependent (Fig. 6, right). The Alpha 300 R microscope showed consistent results with over 90% correctly assigned material up to 200 particles, while for the Apyron, the accuracy declined to 77 ± 11%. Exact numbers can be found in SI, Table S3. For best results, particle numbers per window should be kept below 100 particles when trying to measure small-sized particles.

**Fig. 7:**
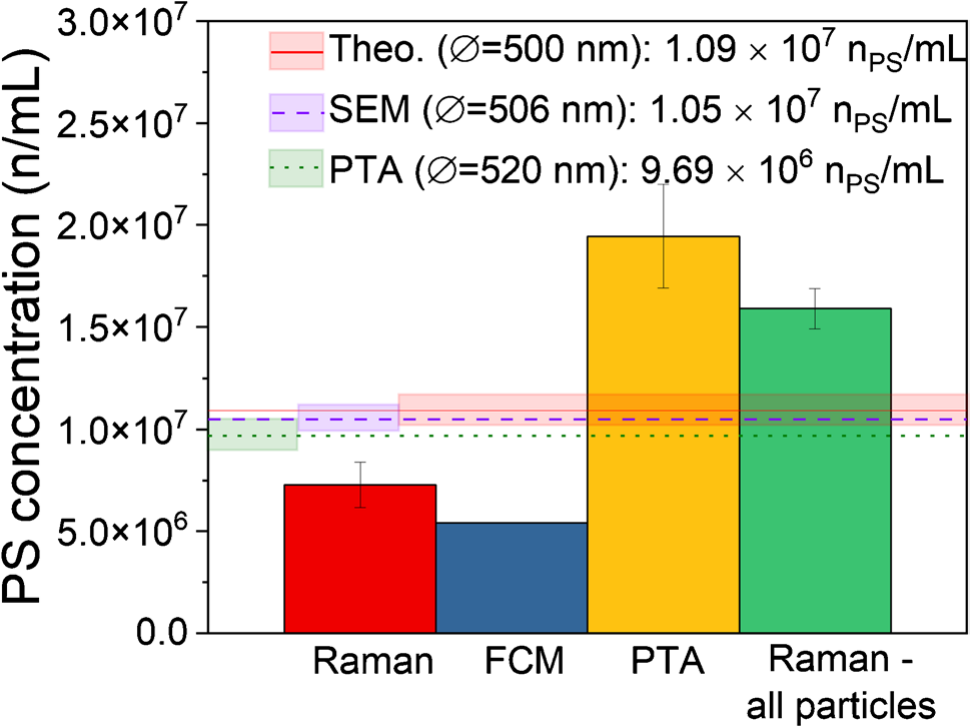
Determined concentration values of prepared PS beads 500 nm-suspension measured with Raman, flow cytometry (FCM), and particle tracking analysis (PTA). Concentration of all particles found with automated Raman measurement shown in green. The theoretically expected value of the suspension was 1.09 × 10^7^ PS particles per mL, when considering a particle size of 500 nm. Additionally, the expected concentrations are given when taking the size determined by SEM and PTA into consideration. The ranges shown include the upper and lower limits of the calculated PS concentration when considering the standard deviation of the particle size

With these limitations in mind, we tested if we were able to automatically measure 500 nm microplastic particles. A suspension of a 500 nm (nominal diameter) PS sphere standard (theoretical 1.09 × 10^7^ particles/mL after dilution) was filtered on an Al-PC filter. To compare the obtained numbers, measurements of the same suspension were performed with particle tracking analysis (PTA) and flow cytometry (FCM) (Fig. 7).

The PTA measurements detected 1.95 × 10^7^ ± 2.55 × 10^6^ particles/mL. Here, it is important to note that PTA detects all particles, independent of their material. The values are, compared to all particles found with the Raman method, quite similar. With FCM, 5.42 × 10^6^ ± 1.77 × 10^4^ plastic particles/mL were detected, which is in good agreement with the Raman results, which detected 7.30 × 10^6^ ± 1.12 × 10^6^ PS particles/mL. Additionally, several other, though in small amounts, plastic particles were detected, such as PE and PET, as well as possible bioorganic materials. SEM imaging revealed interconnected pores and larger openings within the Al-PC membrane (SI, Fig. S8), and single particles were observed partially embedded within these structures. Such pore heterogeneity can result in particle passage through the membrane or retention within pore structures rather than on the surface. Agglomerations of particles on the filter surface were observed with SEM as well, before (dropcast of PS-suspension) and after filtration. FCM also suggests the existence of agglomerated particles (double, triple, quadruple beads, see SI, Fig. S2). In addition to potentially not flushing all particles onto the filter surface, these could be potential reasons for the observed lower detected particle number. Additional deviations from the theoretical particle number arise from uncertainties in the initial suspension concentration. The theoretical particle number concentration is calculated from the nominal sphere diameter, density, and 5 wt% solid content provided by the manufacturer rather than from direct counting. This is a general issue when determining the particle number concentration and is not specific to our method and similar recoveries were reported by other methods for similar sized particles [50, 72]. Due to the uncertainty in the original particle number, it is also difficult to separate the variance due to sample preparation from the variance due to inaccuracy of the analytical method. In Raman-based studies, the focus for concentration valid reference particles has so far been on larger particles and not particles in the submicron size range [73, 74].When incorporating the particle sizes determined by PTA (520 nm ± 13 nm) and SEM (506 ± 10 nm) (%RSD given by manufacturer was 2.2%, which equivalates to 500 nm ± 11 nm), we have a range in concentration from approximately 9.69 × 10^6^ + 6.92 × 10^5^ (533 nm)–7.6 × 10^5^ (507 nm) PS particles/mL (average PTA) to 1.05 × 10^7^ + 7.28 × 10^5^ (496 nm)–6.49 × 10^5^ (516 nm) PS particles/mL (average SEM). This, respectively, would lead to recoveries of 75% ± 12% or 69% ± 12%. The concentrations and their standard deviations of each method were calculated based on the measurement of three different samples (two in the case of FCM) taken from the same suspension. This approach allows an estimation of sample-to-sample variation introduced by pipetting and dilution. For Raman measurements, *TUM-ParticleTyper 2* provides an additional relative error estimation based on bootstrap estimation, which was on average 20.9 ± 0.5%. When incorporating this relative error, the recovery range, using the theoretical value, ranges from 53 ± 8% to 81 ± 12%. When measuring the same filter three times (which in real life application will not be done, due to long measurement times), a deviation of 16% was found between each measurement with an average relative error of 19.3 ± 0.5%. Increasing the measured area or measurement time can reduce this uncertainty to some extent, as discussed previously [67], but this improvement is limited. Combining all three repeated measurements reduces the relative error to 12%. For this filter, the recovery relative to the theoretical value was 86 ± 14% (9.39 × 10^6^ ± 1.51 × 10^6^ PS particles/mL) for the three measurements, which when taking the average recovery determined between different filters of 67 ± 10% into account, showcases the difficulties in obtaining homogenous reference count in suspension as well as a homogenous distribution on the filter. Other factors that may explain the reduced number of detected PS compared to the theoretical value could be unassigned spectra caused by low spectral quality falling below the HQI cut-off. Although this effect is only minor, as visible in the HQI distribution of PS beads for Al-PC in Fig. 2a. As the number of particles was kept to below 30 particles per window (3 ± 5 particles on average), the loss through stage imprecision plays a minor role, but all those factors play into potentially reducing the number of expected PS. Overall, the recovery is primarily limited by (i) aggregations already present in the suspension, (ii) membrane pore heterogeneity leading to embedding and potential loss due to interconnected pores, (iii) uncertainty in nominal suspension concentration, and (iv) statistical variation due to partial surface analysis. The combined influences of these factors explain the observed recovery of 67 ± 10%. When performing the same measurements on Si-0.45 and Anodisc filters, the main reason for the reduced particle number found was in case of the Si-045 filter particle recognition due to issues with the support beams and HQI threshold and for the Anodisc the inability of the automatic system to focus on the filter surface (SI, Fig. S9). Overall, while not achieving the estimated theoretical value, we successfully automatically detected PS beads 500 nm particles on Al-PC filters with a recovery rate calculated from the theoretical value of 67 ± 10% for the Raman measurement. This value exceeds, on average, the suggested recovery rate of 60% recommended in ISO 16094-2 [10]. It should be said, though, that it will be more challenging to apply this recovery rate to real samples, as there are expected to be a multitude of various microplastic types, potentially behaving differently, regarding their recognition. Also, the number of plastic particles in the model system is much higher than the amount found in the water sample.

### Water sample analysis

**Fig. 8:**
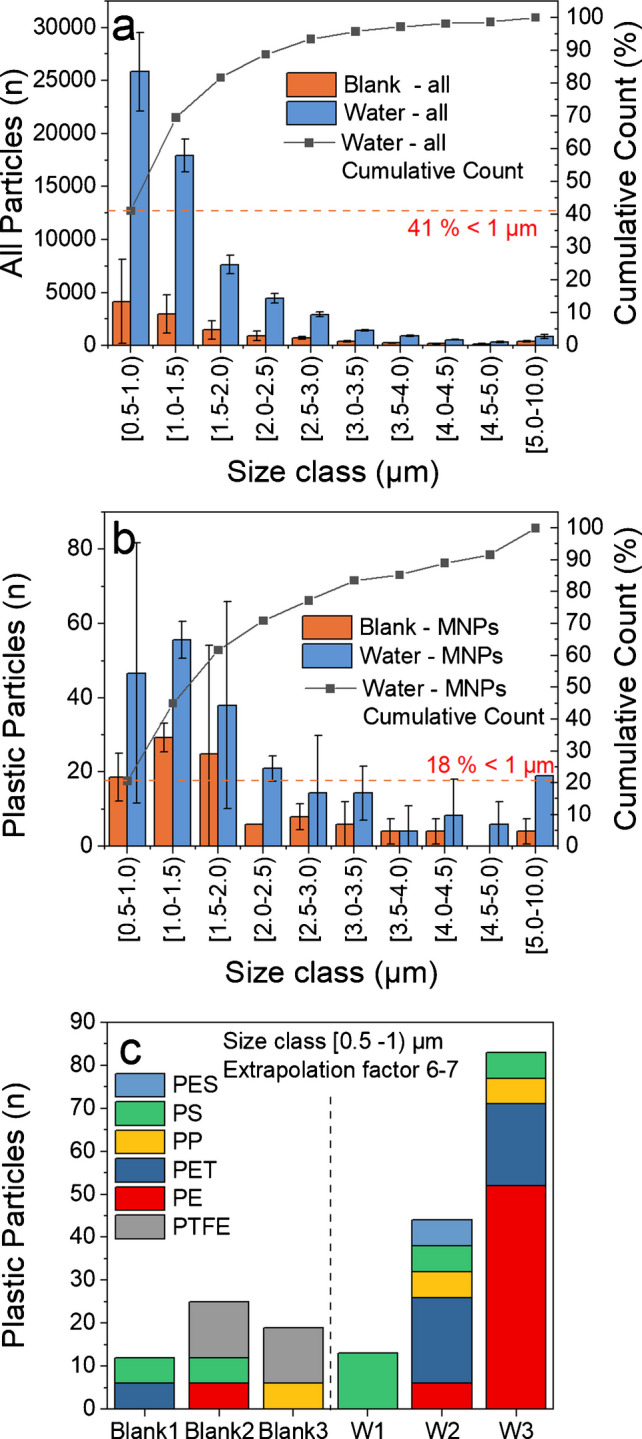
Results from the measurement of three blanks and three water samples (500 mL) filtered on Al-PC filters and extrapolated to the filtration area. Approximately 15% of the filter surface was measured with a field of view sized 170 µm × 170 µm. All particles with a maximum diameter between 0.5 and 10 µm were measured. **a** The size distribution of all measured particles; **b** the size distribution of identified microplastics. **c** The found plastics in the size class between 0.5 and 1 µm

Finally, we applied the knowledge gained to the analysis of a real water sample to evaluate the feasibility of the automated workflow under practical conditions. A water type with comparatively low particle load, resulting in measurable filter surface coverage, was intentionally selected based on preliminary screening, allowing filtration without extensive additional sample preparation. Preliminary tests of other potable water types revealed substantially higher particle loads, in some cases leading to nearly complete coverage of the filter surface (SI, Fig. S10), which would render automated particle-by-particle Raman analysis impractical without prior matrix reduction. This proof-of-principle approach therefore does not cover the full range of possible matrix interferences present in water samples with higher number of (in)organic particles (e.g., industrial wastewater, surface water). For such water types, additional sample preparation step(s) would be required to reduce the number of non-plastic particles and ensure reliable automated analysis. The exposure rate had to be set to a higher value in comparison to the model systems (PS 500 nm beads and PP particles) due to low contrast particles, ensuring the detection of all particles. A window frame of 180 µm × 180 µm with a field of view of 170 µm × 170 µm was chosen. Particles with a maximum diameter within the half-open range [0.5–10 µm) (excluding 10 µm to avoid double-counting in two adjacent size bins) were detected. Each field of view contained, on average, 48 ± 34 particles, which was below the suggested 100 particles per field of view. Measuring with a field of view of 45 µm × 45 µm containing on average 3 ± 4 particles would be closer to a true random sampling. This approach, though, leads to a measurement time three times longer than with the chosen field of view of 170 µm × 170 µm, due to the amount of time it takes for the automated image acquisition and measurement result files saving. In total, for the measurement of three 500 mL water samples, on average 9484 ± 993 particles per sample were automatically measured, taking about 3 days per sample. It is recommended to measure at least 7000 particles to gain a good estimation of microplastic content [75]. A filter surface of 15.1 ± 0.4% (6.6 ± 0.2 mm^2^ from 44.2 mm^2^) was measured, which is much higher than shown in previous studies (e.g., 4% [20], 7.3% [18]). Though when measuring with the smaller field of view with only covering 3% of the filter surface, similar numbers were obtained, though with a higher uncertainty, as with the larger covered filter area (SI, Fig. S11). It has been estimated there to be in 500 mL 62,878 ± 6515 overall particles with 40% found in the size class [0.5–1.0 µm) and decreasing numbers in the following size classes (Fig. 8a). Of those, 0.36 ± 0.13% (227 ± 108 particles per 500 ml) were estimated to be MNPs, with 18.5 ± 8.0% being in the size class[0.5–1.0 µm) (Fig. 8b). While the size distribution shows higher numbers for smaller size classes, fewer MNP particles seem to be found in the size class [0.5–1.0 µm) than expected in comparison to the overall particle count. The expected trend is visible when taking the standard deviation into account (Fig. 8b). Possible reasons might be that the overall MNP count is quite low, with the actual pre-extrapolation count being around 37 plastic particles in total. Split up into the various size classes, the particle count per class is even lower. This is even more prevalent in the case when only about 3% of the filter surface was measured (SI, Fig. S11). For an MNP fraction determined as low as in our case, it is necessary to measure even more particles to gain a stable MNP particle size distribution. Also, the values for each individual sample vary, which can be seen due to the high standard deviations. In the size class [0.5–1.0 µm), PE, PET, PP, PS, and PES were found with various amounts for each water sample. The average overall size classes of  [0.5–10 µm) gave no distinct differentiation between the amount of PE, PET, and PP, all being approximately 20% each of all MNPs found, though again small differences can be seen between each sample (SI, Fig. S12). As the total number of plastic particles is quite low, these distributions should be taken with caution. In the < 1 µm size class, the very low particle numbers and limited analyzed area may result in stochastic variability, which can explain, for example, the apparent differences in PET detection between W1 and W2/W3 or PTFE detection between blanks, despite PET or PTFE being present across all samples or blanks when considering the full size range up to 10 µm (SI, Fig. S12). Although the total particle count in the blank is much lower compared to the samples, the number of microplastic particles detected in the blank is similar to that found in the water samples, albeit the number is slightly lower. Besides plastic particles, the samples also contained biomass and minerals. If their spectra are not contained in the database, it could lead to incorrect assignment during automated material assignment. The program was found to be unable to reliably differentiate between PA spectra and biomass spectra; therefore, the corresponding values were not included. The majority of particles were not Raman active and classified as unknown. Additionally, the spectra might not have been of a high enough quality (e.g., a PET particle with an HQI of 38% was found but excluded in the automatic results generation), or their position was slightly missed due to their size and due to the error of the stage movement. While for the latter, nothing can be done in post-processing, the former could still be included by setting the HQI threshold lower and checking all assigned microplastic spectra by hand and through expert evaluation. This would necessitate additional time for in-person analysis; it is not the desired outcome for a fully automated process, and it increases the risk of overall incorrect assignment of material and non-differentiation between plastic types. The reported microplastic content in water detected with particle-by-particle Raman microspectroscopy analysis down to 1 µm varies between double-digit counts and four-digit counts depending on the water sample type [15, 17, 18, 20]. This overlaps with our results, but as the measurements in this study were performed as proof of principle for the feasibility of detecting nanoplastic particles below 1 µm in a water sample, no conclusions can be drawn about the general content of sub-micrometer plastic particles in water. For this, a wider variety of samples has to be measured.

While this study demonstrates the feasibility of automated Raman analysis of MNPs down to 500 nm, several limitations currently restrict routine application. Particle recognition and size determination are strongly influenced by optical resolution, illumination settings, particle optical properties, and filter surface characteristics, resulting in systematic size overestimation and limited discrimination between sub-micrometer and micrometer-sized particles. Stage positioning accuracy and particle density per measurement window further affect spectral quality and assignment reliability, particularly for particles below 1 µm. In real water samples, low MNP abundance, filter-dependent particle retention, high proportions of non-plastic material, and incomplete spectral databases introduce stochastic variability and uncertainty in size distributions and polymer composition, while long measurement times limit throughput. As a large fraction of particles (40%) was found below 1 µm, measurement time can be substantially reduced by using filters with pore sizes of 1 µm, such as Si filters, when sub-micrometer particles are not of primary interest, also ensuring that submicron-sized particles are not overestimated in size. Additional sample preparation to remove minerals and biomass can further reduce the overall particle number and increase the effective MNP concentration, enabling larger filter areas to be measured within practical time frames (e.g., 20% of filter surface on Si-filter overnight). In addition, the optimized parameters are highly instrument-, magnification objective-, and filter-specific, which currently limits direct inter-laboratory transferability. Overall, these limitations can be mitigated by selecting filter pore sizes according to the targeted size range, improving filter homogeneity and background properties, reducing matrix complexity through sample preparation, adapting illumination and acquisition parameters to each system, increasing the analyzed filter area for low-abundance samples, and integrating advanced image and spectral evaluation approaches, including machine learning.

## Conclusions

The automatic analysis of MNPs down to 500 nm with Raman microspectroscopy in ultrapure water and water samples was successfully achieved. Choosing suitable filter material and illumination parameters was key. Al-PC filter was found to be so far the most suitable filter material due to providing a good contrast between particles and filter surface in darkfield, as well as enhancing the spectral quality to obtain spectra of plastic with a high HQI with automated Raman measurement, which allows automatic material assignment. Several challenges were addressed. The resolution of the image, as well as the chosen illumination settings, plays a major role in the quantification and size determination of the particles. It was found that bright illumination settings resulted in a higher detected number of particles but overestimated the particle size. This can shift detected particles into different size classes, depending on the chosen settings. This is especially the case for particles in the border regions of a size class. Lower illumination settings result in lower detected particle numbers, but more accurate size detection. It was estimated that with balanced illumination, the particles detected in light microscope images, which serve as the basis for particle detection with Raman microspectroscopy, are approximately 0.5 µm larger than their size detected with SEM. The settings must be chosen individually for each Raman microscope system, objective, filter type, and sample type. It was found that over time, minor imprecisions in the stage movement caused some particle positions to be missed. As a result, the Raman measurement was performed adjacent to the particle, yielding only a background spectrum rather than a particle spectrum. Measurements of water samples showed that due to a lot of low contrast particles, the illumination must be set higher than with reference material such as PS beads. The measurement down to 500 nm required for 15% of a 133 mm^2^ filter surface, about 3 days of measurement time. If only data is needed down to 1 µm, it is recommended to use filters with a pore size of 1 µm (e.g., Si filters) to avoid wrong size classification due to overestimation of the size due to the illumination. This also cuts down the measurement time to below 1 day. In this case, Si filters with a 1 µm pore size will be well-suited. The water samples were found to contain a majority of non-plastic material, and for the investigation of other types of waters or more complex samples, more elaborate sample preparations have to be found.

## Supplementary Information

Below is the link to the electronic supplementary material.

Supplementary file1 (PDF 1.45 MB)

## Data Availability

Data will be made available on request from the authors.
