# Supplementary material for: Challenges and solutions in the analysis of micro- and nanoplastics down to 500 nm with automated Raman microspectroscopy: suitable filters, accuracy in the detection, identification, and quantification
Source: Anal Bioanal Chem. 2026 May 29;418(16):5283–302. doi: 10.1007/s00216-026-06567-2 (PMC13424059; doi:10.1007/s00216-026-06567-2)
Supplement: Supplementary file 1 — Supplementary file1 (PDF 1.45 MB) [file 216_2026_6567_MOESM1_ESM.pdf]

Supporting Information (SI)

Isabel S. Jüngling[1], Lucas Schmitt[1], Filippo De Franceschi[2], Paulo A. Da Costa Filho[2], Lei Lei[2], Laureen Coic[3], Nizar Benismail[3], Stephane Dubascoux[2], Mark Ambühl[2], Natalia P. Ivleva[1]*

[1]Technical University of Munich, TUM School of Natural Sciences (NAT, Department Chemistry), Institute of Water Chemistry (IWC), Chair of Analytical Chemistry and Water Chemistry, Lichtenbergstr. 4, 85748 Garching, Germany

[2]Société des Produits Nestlé S.A. Nestlé Research, Route du Jorat 57, Lausanne, Switzerland

[3]Nestle Quality Assurance Center Vittel, 1020 Avenue Georges Clemenceau, 88800 Vittel, France

**Corresponding author: natalia.ivleva@tum.de*

Table S1. Available pore sizes and corresponding manufacturer of the investigated filter types.

Filter	***available pore sizes [µm]***	***Manufacturer***
Si	0.45 [a], 1, 2.5, 5, 8 (up to 18) [b,c]	SmartMembranes GmbH
Al-PC	0.4, 0.8 [a,b]	i3 Membrane
Al-PET	0.8, 3, 5 [b,c]	i3 Membrane
Au-PC	0.1, 0.2, 0.4, 0.8, 3 [b,c]	i3 Membrane, APC
Anodisc	0.02, 0.1, 0.2	Cytiva
Al-Al₂O₃	0.4 [a]	SmartMembranes GmbH
	**a** trial product<br>**b** other pore sizes on request<br>**c** pore density given for each pore size in technical sheets	

Table S2. Filtration rate of the recommended filters. Flow rate was measured three times for each filter (100 mL Milli-Q water previously filtered through 0.2 µm PVDF membrane). Repeatability was quantified using the within-filter standard deviation. Filter-to-filter variability was quantified using the relative standard deviation (%RSD) of individual filter means.

Filter	***Pore size [µm]***	***Pore density given by manufacturer***	***Filtration area [mm²]***	***Mean ± SD [mL/min]***	***%RSD [%]***	***Overall mean ± SD [mL/min]***	***%RSD [%]***
Si	1	40% (4.0 × 10^{7} cm^{-2})	28 (ø 6 mm)	15.9±0.2	1.4	15.3±5.7	37.4
				20.8±0.9	4.2		
				9.3±0.2	1.9		
Al-PC	0.4	1.50 × 10^{8} cm^{-2}	28 (ø 6 mm)	12.5±0.1	0.8	11.1±1.2	10.7
				10.25±0.03	0.3		
				10.6±0.1	0.9		
			132 (ø 13 mm)	46.2±1.1	2.3	40.5±0.3	0.7
				41.5±1.1	2.5		
				33.8±0.6	1.7		

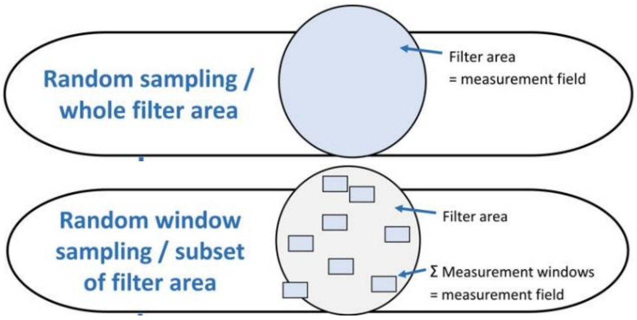

Fig. S1 For analysis with *TUM-ParticleTyper 2*, it is possible to choose between two settings: FullScan, one image over the full filter surface is taken with a lower magnification objective (e.g., 20×), and all particles in the size range 5 µm to 1000 µm are analyzed or if the number exceeds 7000 particles are randomly selected for Raman measurements. WindowSampling developed for the analysis of (microplastic) particles and fibers down to 1 µm generates, depending on entered parameters, a random list of window coordinates at which, for each individual window, an image is taken and the particles in the field of view are analyzed before moving to the next window in the list. The found particle number is, after measurement of all windows, extrapolated to the full given filter surface. This approach allows a random, unbiased measurement of the filter surface and is usually used for particles below 20 µm. It includes a Bootstrap estimation to estimate the relative error. The workflow of the automated analysis with *TUM-ParticleTyper 2* is the following: First, an image is taken in darkfield modus and bright particles are analyzed for position and morphological information by *TUM-ParticleType 2*. This ensures that every image is treated the same way without human bias, which would be the case if the particle recognition of the WITec software was used. An image with generated coordinate points is given to the WITec Particle Scout software, and Raman spectra are measured, and materials are assigned automatically with the TrueMatch Software. The morphological results and material characterization are then combined, and in the case of WindowSampling, the next Window is measured. For a more detailed explanation, refer to Jacob et al. (2023) [1].

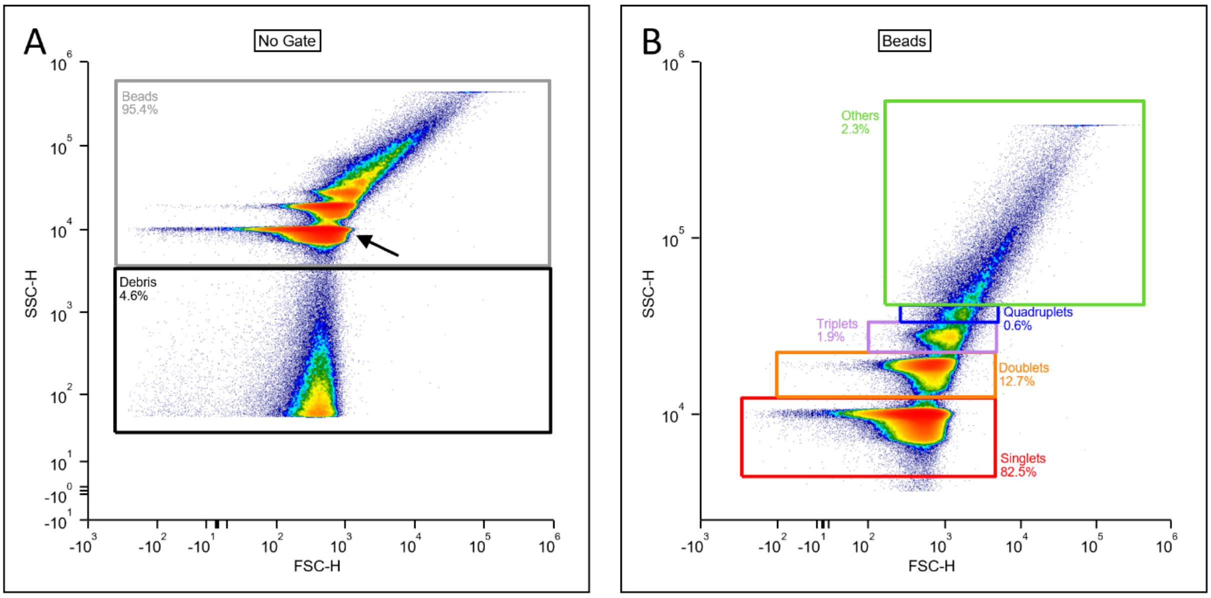

Fig. S2 Flow Cytometry analysis of PS 500 nm beads. (A) 2D Scatter plot showing all detected events. The arrow indicates the main cluster of 500 nm beads (singlets). (B) 2D Scatter plot showing the Beads region from plot A. Singlets, doublets, triplets, quadruplets and others (more than 4 beads together) are gated to show and quantify the percentage of these populations.

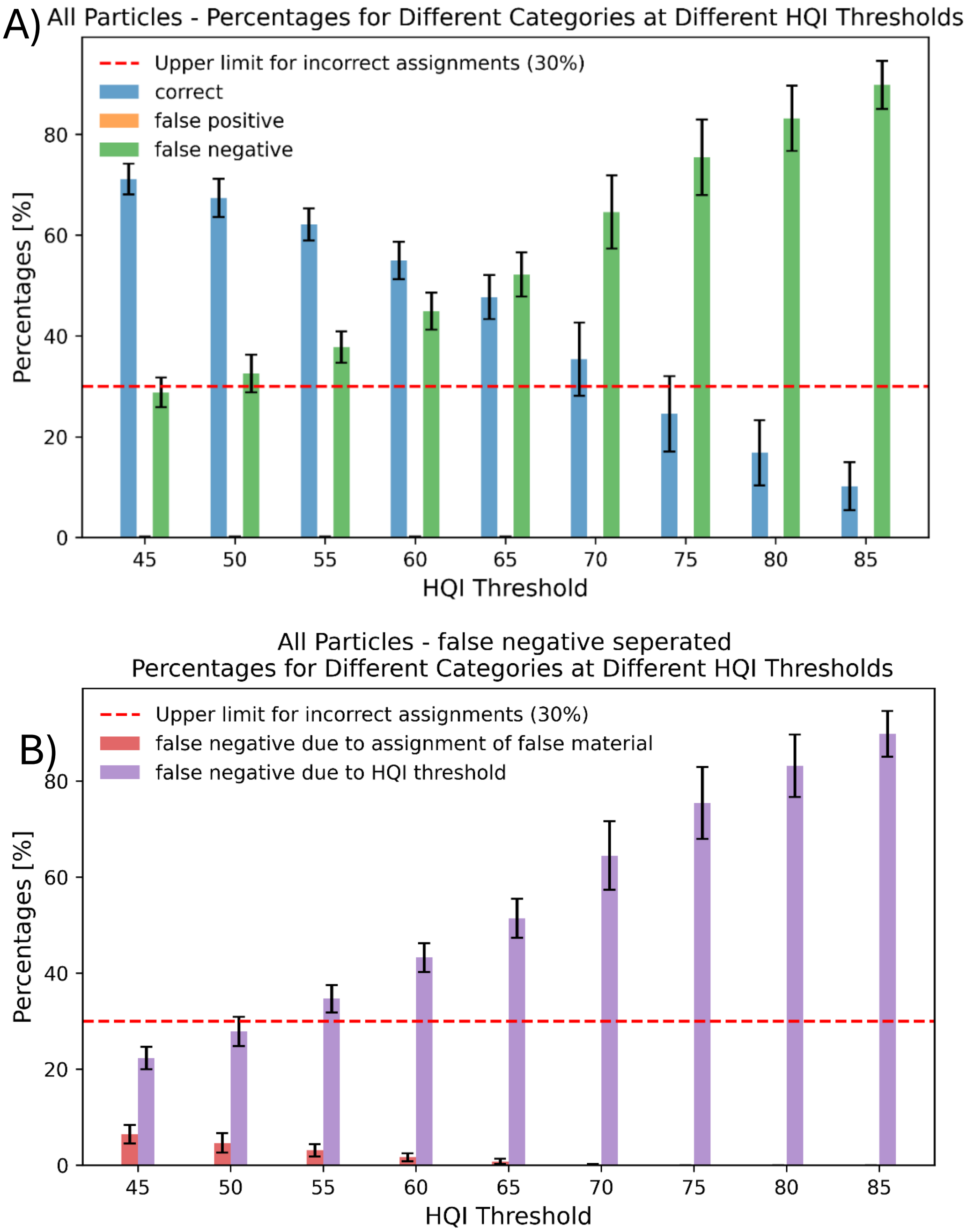

Fig. S3 Effect of the HQI threshold on automated material assignment based on particle spectra of filtered PET, PLA and PS suspensions. The plastic suspensions were created by ultrasonication [2]. Three sets of 500 particle spectra were manually assigned by an operator and compared with the results of the automated assignment algorithm. The top graphic (A) shows the proportion of correctly (blue) and incorrectly (orange and green) assigned particles as a function of the HQI threshold. An error rate below 30% is achieved at an HQI threshold of 45. False-positive assignments (orange; automatic material assignment where no material could be determined by the operator) occurred rarely. Most incorrect assignments were false negatives (green), which are further subdivided in the bottom graphic (B) into false negatives caused by assignment of an incorrect material (red) and false negatives resulting from spectra falling below the selected HQI threshold (purple). Theoretically, the HQI threshold could theoretically be lowered further, discrimination between similar reference spectra (e.g., PA and PMMA) becomes unreliable at lower threshold values.

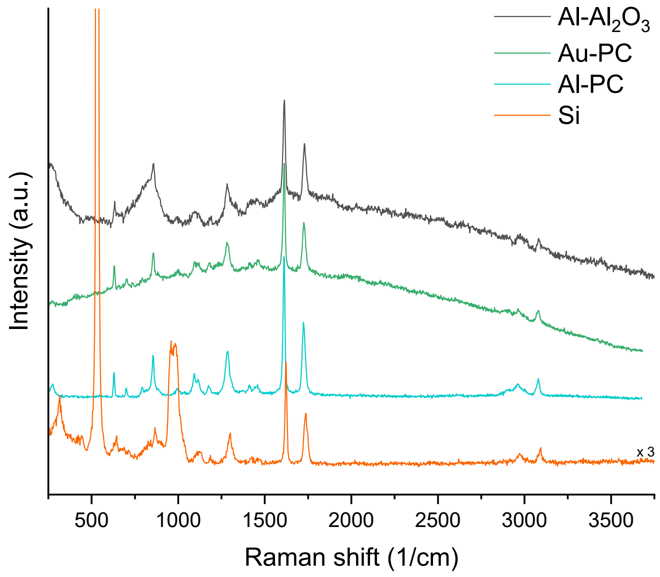

Fig. S4 PET particles (Diam. 6 µm) on different filter types; Al-coated polycarbonate filter (blue) gives the best spectra at similar conditions (integration time 0.5 s, 10 accumulations, laser power 3.5 mW, WITec Alpha 300 R, 100× objective, NA=0.9). PET spectrum on Si-filter multiplied by 3 for better visibility. Silicium bands are visible at 520 cm^{-1} and 920 cm^{-1}.

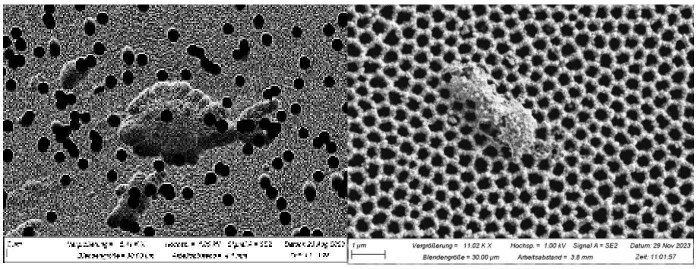

Fig. S5 SEM Image of: Left: Al-PET filter, Right: Al-Al_2O_3-filter. Zoomed in on spots observed with an optical microscope. Additionally, visible: random (Al-PET) and grid (Al-Al_2O_3) structure of pores.

Anodisc with PS-500 nm beads

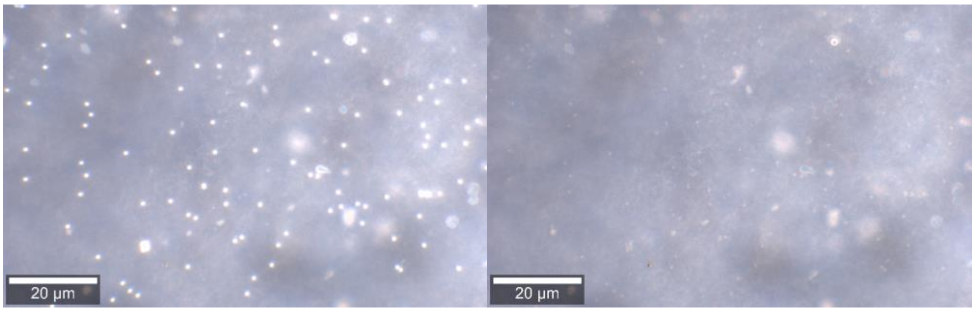

Fig. S6 Left: PS-beads focused by hand. It is possible to differentiate between filter surface structure and PS-beads but overlap exists. Right: Automated focus. PS-beads are not clearly visible anymore. Also, the surface structure is more prevalent.

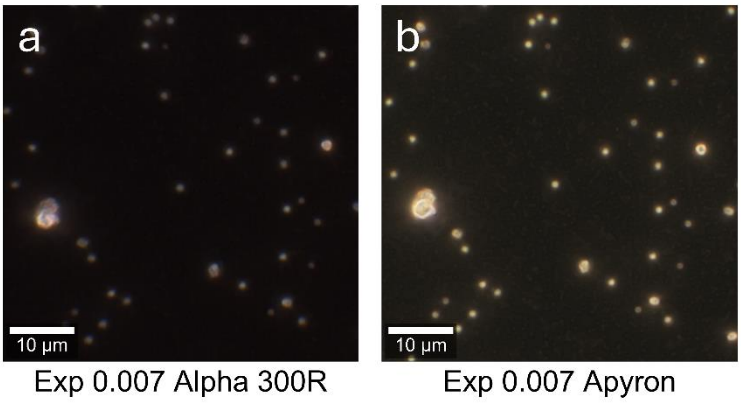

Fig.S7 a & b: Images of PS-beads 500 nm taken with two microscopes at the same nominal exposure of 0.007. Brightness differs between microscopes.

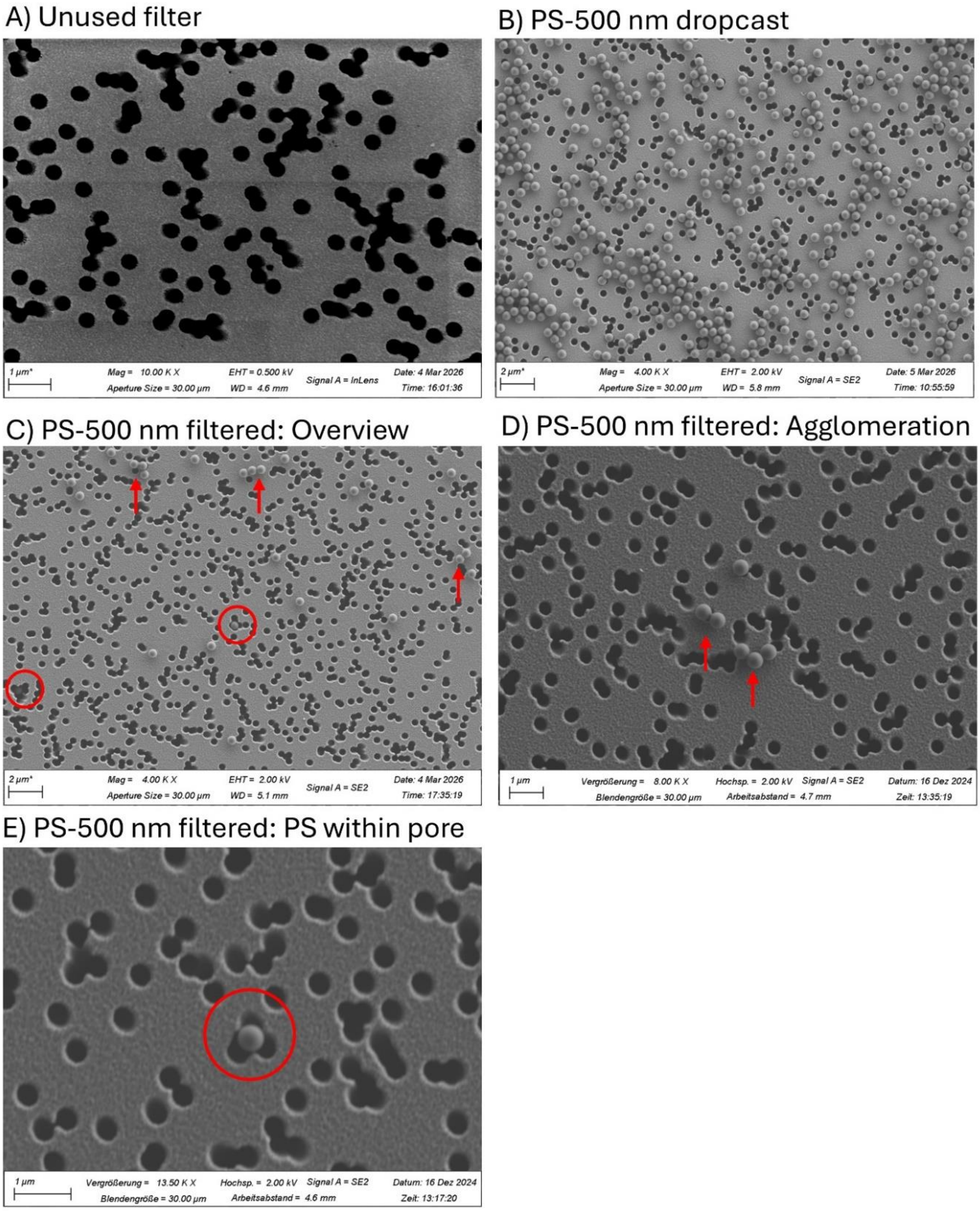

Fig. S8 SEM images of A) unused Al-PC filter. Pores forming larger holes due to accumulation in one place. B) PS-500 nm beads drop cast on unused Al-PC filter. C) PS-500 nm beads filtered on Al-PC filter giving an overview over agglomerated beads and beads embedded in filter pores. Filter used for Raman measurements. D) PS-500 nm beads filtered on Al-PC filter showing agglomeration at higher magnification. E) PS-500 nm beads filtered on Al-PC filter showing a bead embedded in a pore at higher magnification.

Table S3 For different microscopes mean values of the number of correctly assigned material spectra in dependence on the number of particles already measured. The rate of decrease in accuracy over time is device-dependent. Most particles were below 2.5 µm in diameter, with approximately 40 % below 1 µm.

Microscope	Particle number measured<br>Correct material assigned [%]			
	50	100	150	200
**Mean values (*Alpha*)**	93 ± 6%	94 ± 6%	94 ± 5%	91 ± 5%
**Mean values (*Apyron*)**	90 ± 4%	88 ± 5%	83 ±7%	77 ± 11%

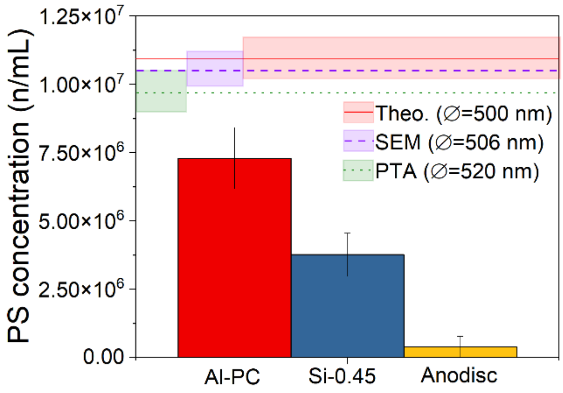

Fig. S9 Determined concentration values of prepared PS-beads 500 nm suspensions measured with Raman microspectroscopy on three different filter substrates. The calculated PS concentrations, depending on the average determined particles size (theoretical manufacturer given, SEM and PTA) and their ranges are shown.

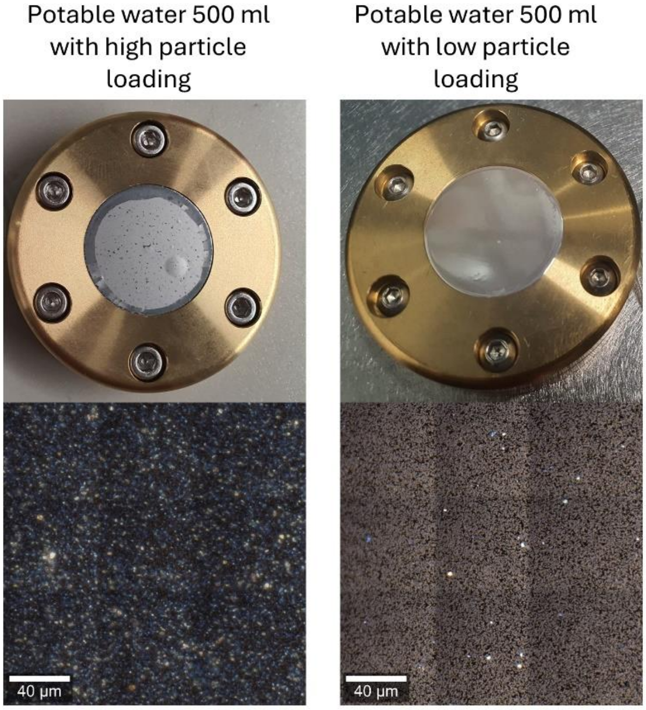

Fig.S10 Camera images and light microscope images of filters obtained from preliminary screening of different water samples prior to selection of the final test matrix. One water type exhibited very high particle coverage, leading to overlapping particles and impractical particle-by-particle Raman analysis without additional sample preparation. These observations motivated the selection of a lower-load water sample for the feasibility study presented in the main manuscript.

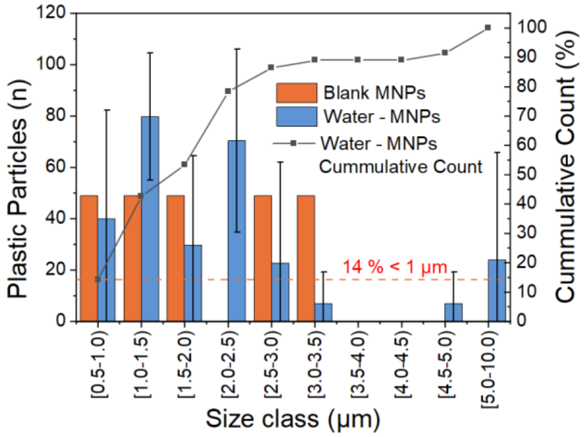

Fig. S11 MNPs size distribution for sample measured with a measurement window of 55 µm × 55 µm and extrapolated to filtration surface. Approximately 3% of the filter surface was measured. All particles with a maximum diameter between 0.5 µm and 10 µm were measured.

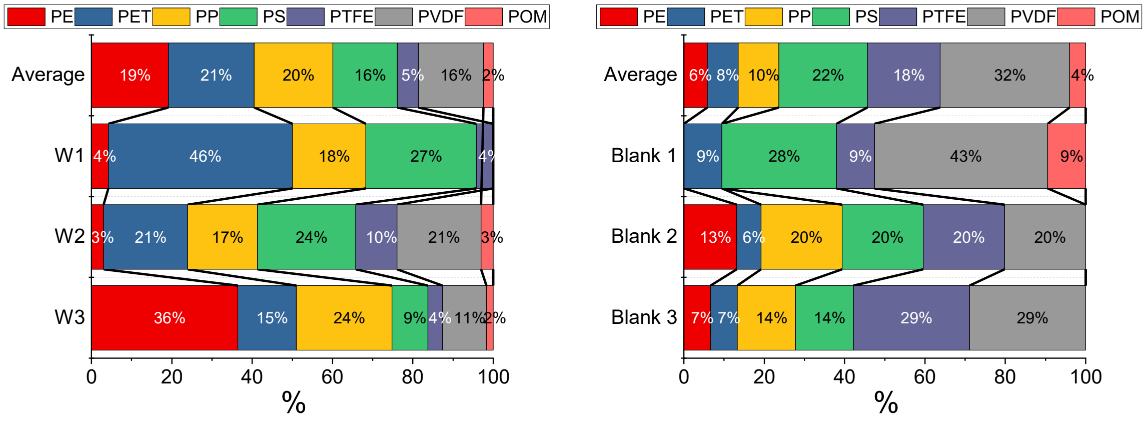

Fig. S12 Total MNPs content given in percentage for the size class [0.5 – 10[µm for water samples and blanks.

References

1. Jacob O, Ramírez-Piñero A, Elsner M, Ivleva NP. TUM-ParticleTyper 2: automated quantitative analysis of (microplastic) particles and fibers down to 1 µm by Raman microspectroscopy. Anal Bioanal Chem. 2023;415:2947–61. doi:10.1007/s00216-023-04712-9.
2. Esch E von der, Kohles AJ, Anger PM, Hoppe R, Niessner R, Elsner M, Ivleva NP. TUM-ParticleTyper: A detection and quantification tool for automated analysis of (Microplastic) particles and fibers. PLoS One. 2020;15:e0234766. doi:10.1371/journal.pone.0234766.
